# Conditions in Home and Transplant Soils Have Differential Effects on the Performance of Diploid and Allotetraploid *Anthericum* Species

**DOI:** 10.1371/journal.pone.0116992

**Published:** 2015-01-21

**Authors:** Lucie Černá, Zuzana Münzbergová

**Affiliations:** 1 Department of Botany, Faculty of Science, Charles University, Prague, Czech Republic; 2 Institute of Botany, Academy of Sciences, Průhonice, Czech Republic; Jyväskylä University, FINLAND

## Abstract

Due to increased levels of heterozygosity, polyploids are expected to have a greater ability to adapt to different environments than their diploid ancestors. While this theoretical pattern has been suggested repeatedly, studies comparing adaptability to changing conditions in diploids and polyploids are rare. The aim of the study was to determine the importance of environmental conditions of origin as well as target conditions on performance of two *Anthericum* species, allotetraploid *A. liliago* and diploid *A. ramosum* and to explore whether the two species differ in the ability to adapt to these environmental conditions. Specifically, we performed a common garden experiment using soil from 6 localities within the species’ natural range, and we simulated the forest and open environments in which they might occur. We compared the performance of diploid *A. ramosum* and allotetraploid *A. liliago* originating from different locations in the different soils. The performance of the two species was not affected by simulated shading but differed strongly between the different target soils. Growth of the tetraploids was not affected by the origin of the plants. In contrast, diploids from the most nutrient poor soil performed best in the richest soil, indicating that diploids from deprived environments have an increased ability to acquire nutrients when available. They are thus able to profit from transfer to novel nutrient rich environments. Therefore, the results of the study did not support the general expectation that the polyploids should have a greater ability than the diploids to adapt to a wide range of conditions. In contrast, the results are in line with the observation that diploids occupy a wider range of environments than the allotetraploids in our system.

## Introduction

Polyploidy is a major factor driving angiosperm evolution [1]. Polyploidization has been shown to have consequences for a wide range of plant traits. These include various physiological (e.g., [2], [3], [4]) and size-related traits (e.g., [5], [6]), differences in flowering phenology and flower morphology (e.g., [7], [8], [9]) and different habitat requirements, distributions and local and landscape dynamics (e.g., [10], [11], [12], [13], [14]). However, there are also studies that indicate a lack of differences between diploids and polyploids in the above-mentioned traits (e.g., [15], [16], [17], [18], [19]).

The differences in species habitat requirements and their distributions are often related to the increased ability of polyploids to adapt to a wide range of conditions due to their higher degree of heterozygosity and overall genetic diversity ([3], [20]). In addition, if the polyploids originated via allopolyploidization, i.e., by hybridization of two different species, they will also have increased heterozygosity and overall genetic diversity thanks to their hybrid origin [21]. These processes are commonly used as explanations of the fact that diploids typically occupy narrower ranges and fewer habitat types than polyploids ([12], [14], [22]). However, in a previous study dealing with two congeneric species, the allotetraploid *Anthericum liliago* and the diploid *Anthericum ramosum*, we Černá and Münzbergová [23] have shown that the diploid species occupies a wider range of environments in the study region than the closely related allotetraploid species. This pattern could, in fact, suggest that the diploid species has a greater ability to adapt to different environmental conditions than the tetraploid species. Theoretically, this could be linked to the fact that the tetraploid species occurs at the edge of its range in the study region, and populations at the edge of their ranges are known to have lower genetic diversity and thus a lower ability to adapt to different environmental conditions ([24], [25], [26]). In addition, the differences might be caused by the fact that the allotetraploid species might be relatively new and therefore has not yet been able to adapt to different conditions [27].

In our previous field study [23], we compared the full life cycle of the two species in their natural habitats. The results suggested that the population dynamics between the two species are more similar when they occur in the same environmental conditions than between populations of the same species occurring in different environment types. Because the study was only observational, we cannot determine whether the differences are the result of adaptation of the plants to the local environment or whether the current environmental conditions determine the growth of the plants in each location. Also, we cannot exclude the possibility that contrasting environmental distribution of the two species may not reflect adaptation but chance colonization events [28]. Detecting local adaptation and possible limits to species distribution requires reciprocal transplant experiments in which plants from different origins are cultivated in those different conditions ([29–31]).

Reciprocal transplant experiments to study local adaptation can be performed by either transplanting the plants between different localities in the field or by simulating the environmental conditions in a common garden [29]. While the former provides more realistic results as it captures all of the characteristics of the localities, the latter is a much better approach for understanding specific driving factors ([32, 33]). Common garden experiments are also the only option when dealing with species that are rare and/or occur in protected habitats.

A wider distribution in diploids compared to closely related polyploids was also previously found for *Mercurialis annua* [31]. Reciprocal transplant experiments have demonstrated that the wider distribution of diploids is not due to local adaptation but to higher diploid fitness across all environments [31]. This study is one of the very few to use reciprocal transplant experiments to study local adaptation in a diploid-polyploid system. In fact, the other three studies that performed such a comparison ([34–36]) found neither major differences in the degree of local adaptation between the cytotypes nor superior overall fitness of one of the types.

The aim of the study was to determine the importance of environmental conditions in which they grow as well as of the conditions from which they originated for performance of two *Anthericum* species, allotetraploid *A*. *liliago* and diploid *A*. *ramosum* and to explore whether the two species differ in the ability to adapt to these environmental conditions. To fulfill the aim, we performed a common garden experiment using the allotetraploid *A*. *liliago* and diploid *A*. *ramosum*. We simulated the two environments in which the species occur, i.e. open and forest environments. We used individuals from the populations of both species from the open environment and grew them in simulated open and forest environments. In addition, we used forest populations of *A*. *ramosum* and also grew them in both environments; *A*. *liliago* does not occur in forests. In this way, we could compare the performance of both cytotypes in the different environments and assess whether the diploids form multiple locally adapted types or exhibit better performance in all of the environments regardless of their origin. By performing the experiment in common garden conditions, we specifically focused on the effect of shading and soil on plant performance. By growing all the plants in their soils of origin as well as in the other soils, we could also explore whether the plants are adapted to their home conditions.

We predicted that the target environment will have significant effect on plant performance. We also predicted that not only the target environment but also the environment of origin will have significant effect on plant performance. Finally, we predicted that response to the environment of origin and the target environment will differ between species. Specifically, we expected that performance of the diploid *A*. *ramosum* from the open environment will not be strongly reduced in simulated forest environment, whereas the allotetraploid *A*. *liliago* will perform worse in the simulated forest environment in which it does not occur in the nature. In addition, we expected that populations of *A*. *ramosum* from different environments will show signs of local adaptation.

## Methods

Collection of the seed material and soil samples for the experiment was permitted by the Regional Unit for Nature Protection for Central Bohemia, Czech Republic.

### Study species

*A*. *liliago* L. and *A*. *ramosum* L. (Asphodelaceae) are closely related, long-lived perennial herbs typical of dry grasslands and undergo frequent generative as well as clonal reproduction. *A*. *liliago* is an allotetraploid (2n = 60), and *A*. *ramosum* is one of its diploid (2n = 30) progenitors [37]. The second progenitor of *A*. *liliago* is not known, but one possibility is that it is a diploid *A*. *liliago*, which has been reported from the Alps and Denmark. The second possible candidate is a Spanish endemic, *A*. *baeticum*, that is currently not sympatric with the other taxa [37]. The species are able to produce triploid hybrids, and triploid populations are known from Scandinavia [37]. However, the existence of triploids in nature in the study region, the Czech Republic, has not been confirmed ([23, 38]). The distribution of both species in Europe covers an area from Spain across France and central Europe to the Balkans and Ukraine. In the north, the distribution reaches southern Sweden and the Baltic states. In the Czech Republic, the diploid *A*. *ramosum* can be found both in Bohemia (western) and Moravia (eastern). The eastern boundary of the distribution of allotetraploid *A*. *liliago* is in central Bohemia. Outside Europe, *A*. *ramosum* the distribution range reaches up to Caucasus and Turkey. In contrast, the only country hosting *A*. *liliago* outside Europe is Turkey (http://e-monocot.org).

In the Czech Republic, the two species usually occur separately. Allotetraploid *A*. *liliago* prefers open, sunny, stony slopes and rocks with southern exposure. Diploid *A*. *ramosum* occupies sunny hillsides, often on a calcareous substrate and dry open forests. The two species do co-occur on some of the open, sunny slopes.

### Study localities

**Figure 1:**
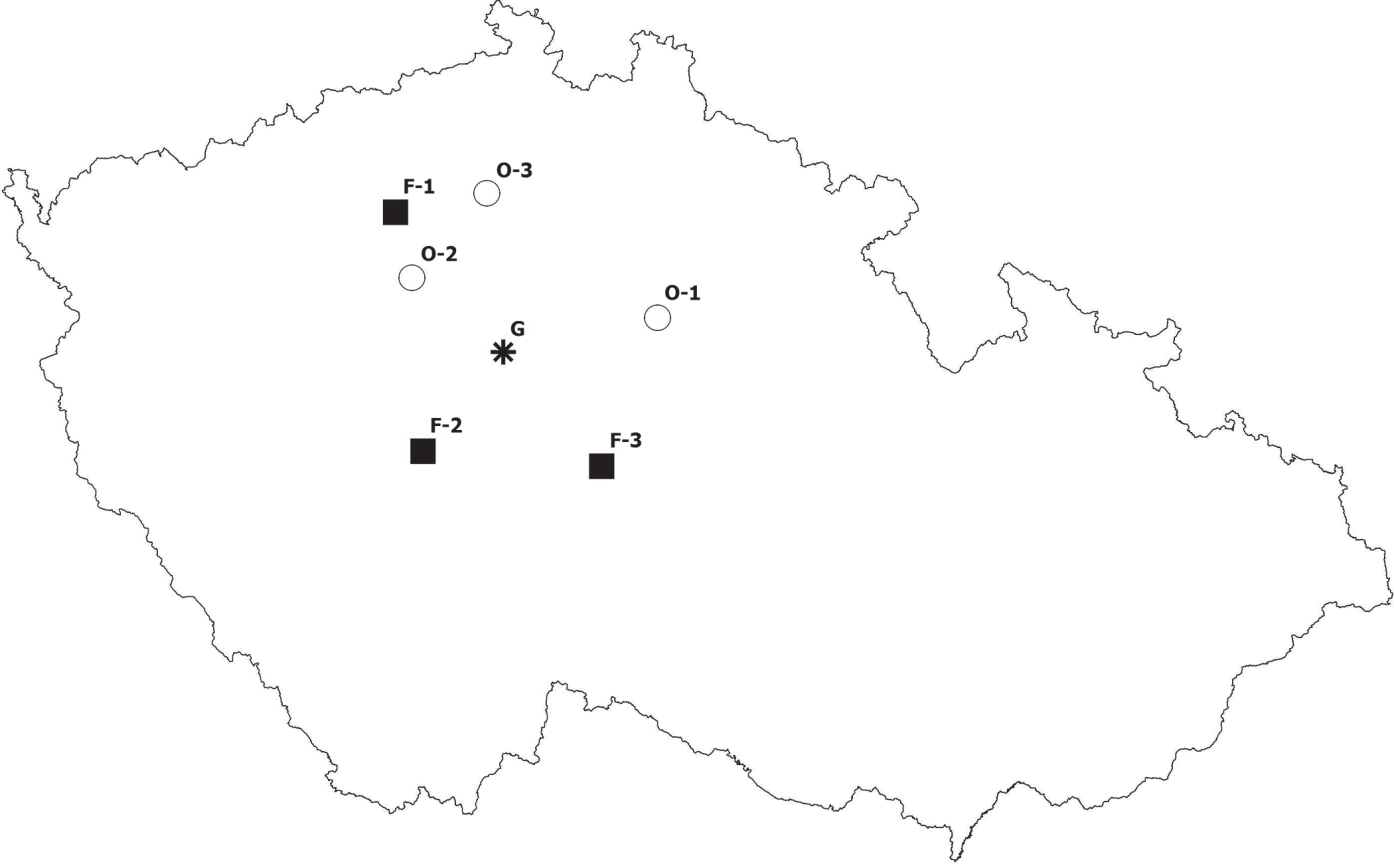
A map of the study localities in the Czech Republic, Europe. Circles indicate the open localities (*A*. *liliago* as well as *A*. *ramosum*), and squares indicate the forest localities (*A*. *ramosum* only). G indicates the location of the experimental garden.

To compare the growth of *A*. *liliago* and *A*. *ramosum*, we selected 3 localities in open environment where the two species co-occur in the Czech Republic. To contrast the growth of *A*. *ramosum* in both environments, 3 additional forest localities were chosen. From each of these 6 localities, we sampled seeds of populations of each *Anthericum* species occurring at the given locality. This resulted into 9 populations in total (3 populations of *A*. *liliago* from open localities, 3 populations of *A*. *ramosum* from open localities and 3 populations of *A*. *ramosum* from forest localities). Positions of these localities are shown in Fig. 1.

To characterize the soil from the localities, we collected 5 samples per locality and analyzed the pH in H_2_O and the contents of phosphorus, total nitrogen and total carbon. The samples from each locality were taken from a depth of 20 cm, and the sampling sites were distributed throughout the locality. Soil samples were sieved through a 2-mm sieve and prepared for chemical analyses [39]. We determined the actual and exchangeable pH using deionized water and a 0.1 M solution of KCl as the extractable solutions, respectively. Available P was determined using the photometric methods [40]. Total C and N contents were determined using a CHN analyzer ([41], Carlo Erba NC 2500).

In addition, five soil monoliths (100 cm^3^) were collected at each locality from the same sampling points. These monoliths were used to estimate the water holding capacity of the soil, which was measured as the amount of water that remained bound in the soil monolith after standing on constantly wet filter paper for 24 hours and expressed as the amount of water retained per gram of dry soil [42].

### Common garden experiment

The performance of *A*. *liliago* and *A*. *ramosum* from all 9 populations was studied for 2 seasons (2007 and 2008). To simulate the local environments in the common garden, we collected soil from each of the 6 localities (resulting into 6 soil types). Soil mixtures from the same 5 sampling points were used to both study soil chemistry and in the common garden experiment. Soil from each locality was thoroughly mixed and poured into 90 plastic pots (10 × 10 × 10 cm). Seeds from 10 randomly selected maternal plants from each study population were collected in the summer of 2006. In the winter of 2006, three to six seeds were sown into each pot. Seeds from each maternal plant were planted in one pot for each soil type. Together, 540 pots (9 plant populations × 6 soil types × 10 maternal plants) were used in the experiment. Except for natural rainfall, the pots containing soil from the open localities were only watered under severe drought conditions. The pots from the forest localities were watered daily, and the soil from the forest localities was shaded. Shading was achieved with a green garden shading net that transmitted 35% of the incident radiation. This level of shading is comparable to that in the forests (shading in the natural forest localities reduces light by 63.3% ± 1.95, a mean for all localities with 10 measurements per locality).

In the spring of 2007, all the germinated seedlings were removed from all pots except one. The seed germination was very high with almost 100% germinating in all pots. As a result, the data on seed germination were not analyzed. The aboveground size of the plants (length and number of leaves) was measured in July, 2007 and 2008. We also recorded whether the plants flowered. In August of 2008, the plants were harvested, and their above-ground biomass was dried to a constant weight and weighed. Because the data for leaf length and number of leaves provided similar results as with plant biomass, only the results based on biomass are presented here.

We measured plant performance as plant aboveground biomass and assumed this value to be a proxy for plant fitness. It has been repeatedly demonstrated that aboveground biomass is positively correlated with flowering intensity and seed production (e.g., [43–48]). In addition, the studied species not only reproduce generatively but also show extensive vegetative reproduction [23], and the intensity of clonal reproduction is also a function of plant size ([49], [50]). Plant aboveground biomass is thus expected to be a suitable proxy for plant fitness in these species. While it would be useful to also have data on belowground biomass of the plant, such data are unfortunately not available for technical reasons.

### Data analysis

To understand the differences in chemical composition between the different soils used in the experiment, we tested the effect of environment type (open and forest) and locality (6 localities) nested within environment type on the properties of single chemicals and water-holding capacity using analysis of variance (ANOVA, Table 1).

**Table 1:** The effect of environment type and locality nested within environment type on single soil characteristics (pH in H_2_O, contents of phosphorus, nitrogen, carbon, carbon to nitrogen ratio and water holding capacity).

				Open			Forest		
	Type	Locality		O-1	O-2	O-3	F-1	F-2	F-3
pH (H_2_O)	F = 80.05	F = 7.78	Mean	8.1	8.1	8.4	7.8	5.9	6.7
	p < 0.001	p = 0.001	SE	0.04	0.02	0.09	0.03	0.28	0.04
				ab	ab	a	b	c	d
P (mg/kg)	F = 27.77	F = 3.25	Mean	5.8	6.6	5.2	6.7	7.9	8.3
	p < 0.001	p = 0.049	SE	0.36	0.28	0.32	0.28	0.69	0.61
				ab	bc	a	abcd	cd	d
N (%)	F = 41.74	F = 10.30	Mean	0.3	0.3	0.1	0.5	0.2	0.6
	p < 0.001	p < 0.001	SE	0.02	0.01	0.01	0.02	0.04	0.05
				a	a	b	c	a	c
C (%)	F = 4.36	F = 4.37	Mean	6	7.1	3.5	8.4	3.8	8.1
	p = 0.043	p = 0.019	SE	0.23	0.16	0.21	0.46	0.67	0.88
				a	ab	c	b	c	b
C/N	F = 33.32	F = 32.78	Mean	22.2	25.5	67.7	17	15.6	13.9
	p < 0.001	p < 0.001	SE	1.27	0.64	8.20	0.34	0.79	0.66
				a	a	b	a	a	a
Water holding capacity (%)	F = 0.19	F = 23.68	Mean	42	43.7	27.6	32.2	29	49.5
	p = 0.666	p < 0.001	SE	1.39	0.68	0.73	1.86	2.69	2.05
				a	ac	b	b	b	c

The mean and standard error of the mean values of the soil chemical characteristics at the studied localities are also shown. Localities marked by the same letter are not significantly different from each other. Significant values are in bold. The codes O-1 to F-3 are locality codes and correspond to codes used in Figures.

All of the analyses of data from the garden experiment were separated into two parts, i.e., 1) the comparison of the populations of *A*. *liliago* and *A*. *ramosum* from the open environments and 2) the comparison of *A*. *ramosum* from the open environments with *A*. *ramosum* from the forests. As *A*. *liliago* does not form large forest populations and could not be studied in that environment, the study design was not fully factorial. To adjust for the fact that the same data were always used for two independent tests, the conventional α level was reduced from 0.05 to 0.025 (cf. [15]).

The dependent variable was the aboveground biomass at the end of the experiment. Aboveground biomass was square root transformed to achieve normality and tested using ANOVA. The second dependent variable was flowering state (yes/no), which was tested using logistic regression. The differences in flowering were only compared for *A*. *ramosum* in the two environments as less than 1% of the *A*. *liliago* individuals flowered during the experiment.

When comparing *A*. *ramosum* from the two environments, we tested the effect of the target environment (simulated open or forest environment), target soil (i.e., soil where the plants were experimentally planted; 6 soil types, one from each locality), environment of origin (open or forest environment) and locality of origin of the transplanted plants (3 open and 3 forest localities). The target soil was nested within the target environment, and the locality of origin was nested within the environment of origin. All of the factors were treated as fixed. For tests of significance, we used the change in mean deviance, the quasi F-ratio [51]. Specifically, we tested target environment against target soil (Table 2) and environment of origin against locality of origin (Table 2). Then, we tested the target environment × environment of origin interaction against the target soil × locality of origin interaction. Target soil, locality of origin and target soil × locality of origin interaction were all tested against the residuals (Table 2). A significant target environment × environment of origin interaction would indicate adaptation to open and forest environments. Similarly, a significant target soil × locality of origin interaction would indicate adaptation at the population level.

**Table 2:** The determinants of plant performance in the experiment.

A) *A*. *ramosum* from open and forest environments					
Aboveground biomass					
	Error	df	df error	quasi F	p
Target environment (TE)	TS	1	4	0.048	0.837
Target soil (TS)	Residuals	4	302	15.109	< 0.001
Environment of origin (EO)	LO	1	4	0.018	0.901
Locality of origin (LO)	Residuals	4	302	9.205	< 0.001
TE × EO	TS × LO	1	24	0.046	0.833
TS × LO	Residuals	24	302	1.501	0.065
B) *A*. *ramosum* from open and forest environments					
Flowering					
	Error	df	df error	quasi F	p
Target environment (TE)	TS	1	4	0.422	0.551
Target soil (TS)	Residuals	1	302	7.434	0.007
Environment of origin (EO)	LO	4	4	0.027	0.998
Locality of origin (LO)	Residuals	4	302	7.866	< 0.001
TE × EO	TS × LO	1	24	0.051	0.824
TS × LO	Residuals	24	302	1.525	0.057
C) *A*. *ramosum* and *A*. *liliago* from open environments					
Aboveground biomass					
	Error	df	df error	quasi F	p
Target environment (TE)	TS	1	4	0.239	0.651
Target soil (TS)	Residuals	4	312	23.920	< 0.001
Species	Residuals	1	312	8.592	0.004
Locality of origin (LO)	Residuals	2	312	28.211	< 0.001
Species × LO	Residuals	2	312	4.278	0.015
TE × species	TS × species	1	4	2.786	0.170
TE × LO	TS × LO	2	8	0.697	0.526
TS × species	Residuals	4	312	1.609	0.172
TS × LO	Residuals	8	312	1.110	0.356
TE × species × LO	Residuals	1	312	0.001	0.996
TS × species × LO	Residuals	2	312	0.001	0.999

A) The effects on aboveground biomass when comparing *A*. *ramosum* from open and forest environments. B) The effect on flowering probability when comparing *A*. *ramosum* from open and forest environments. C) The effects on aboveground biomass when comparing *A*. *ramosum* and *A*. *liliago* from the open environments. Significant values are in bold.

To test the effect of specific soil conditions on plant growth, we replaced target soil and locality of origin with specific values characterizing soil chemistry–specifically, values for pH and the contents of phosphorus, nitrogen, carbon, carbon to nitrogen ratio and water holding capacity–at both the localities of origin and the target soils. We also tested the interactions between soil chemistry of the target soil and the locality of origin (Table 3). The test was performed as described above, and significant soil chemistry values were selected using step-wise regression in both directions in S-Plus (2000).

**Table 3:** The effect of specific soil characteristics of the target soil and soil of origin on plant performance in the experiment.

A) *A*. *ramosum* from open and forest environments						
Aboveground biomass						
	Error	df	df error	F	p	direction
Target environment (TE)	TS	1	4	0.048	0.837	
Environment of origin (EO)	LO	1	4	0.018	0.901	
TE × EO	TS × LO	1	24	0.046	0.833	
Origin pH	Residuals	1	325	3.099	0.079	
Origin P	Residuals	1	325	1.375	0.242	
Origin N	Residuals	1	325	31.535	< 0.001	-
Origin C	Residuals	1	325	0.064	0.801	
Target P	Residuals	1	325	11.330	0.001	+
Target N	Residuals	1	325	26.174	< 0.001	+
Target C	Residuals	1	325	17.045	< 0.001	+
Origin C/N * target C/N	Residuals	1	325	13.877	< 0.001	
B) *A*. *ramosum* from open and forest environments						
Flowering						
	Error	df	df error	F	p	direction
Target environment (TE)	TS	1	4	0.422	0.551	
Environmentof origin (EO)	LO	1	4	0.027	0.998	
TE × EO	TS × LO	1	24	0.051	0.824	
Origin pH	Residuals	1	325	8.185	0.004	-
Origin P	Residuals	1	325	5.550	0.018	+
Origin C	Residuals	1	325	13.936	0.000	-
Target pH	Residuals	1	325	27.107	<0.001	+
Target P	Residuals	1	325	0.068	0.795	
Origin C/N * target C/N	Residuals	1	325	18.340	<0.001	

A) The effects on aboveground biomass when comparing *A*. *ramosum* from open and forest environments. B) The effect on flowering probability when comparing *A*. *ramosum* from open and forest environments. The table corresponds to Table 2A and B with the effect of target soil and locality of origin replaced by the specific habitat characteristics and their interactions; only the characteristics selected using step-wise repression are shown. The between species comparison is not shown as no habitat characteristics had any significant effect. Significance values are in bold, and direction indicates the direction of the significant effects if applicable (for the interactions, see Results).

When comparing *A*. *ramosum* and *A*. *liliago* from the open environments, we characterized the target environment (simulated open and forest) and the target soil (i.e., the soil where the plants were planted experimentally; 6 soil types, one from each locality)). Further, we tested the effect of species and the locality of origin of the transplanted plants (3 open localities). The target soil was nested within the target environment, and all of the factors were considered to be fixed. We tested target environment against target soil (Table 1). In contrast, both species and locality of origin were tested against the residuals as these two variables were combined in a factorial fashion (both species originated from all open localities). We also tested the interaction between species and locality of origin. Furthermore, the model contained a target environment × species interaction, target environment × locality of origin interaction, target soil × species interaction and target soil × locality of origin interaction. The interactions with the target environment were tested against their respective interactions with the target soil, and the interactions with the target soil were tested against the residuals. We also tested the triple interaction of target soil or target environment × species × locality of origin (Table 1). An interaction between species and locality of origin and/or between species and target environment or target soil would indicate that the two species differ in their ability to respond to various environment conditions.

To test the effect of specific soil conditions on plant aboveground biomass, we replaced target soil and locality of origin with specific values describing soil chemistry–specifically, values for pH and the contents of phosphorus, nitrogen, carbon, carbon to nitrogen ratio and water holding capacity–of both the localities of origin and the target soils. We also tested the interactions between the soil chemistry of the target soils and the locality of origin and the interaction of the species with the target soil and the soil characteristics of the locality of origin (Table 3). The test was performed as described above. The significant soil chemistry values were selected using step-wise regression in both directions in S-Plus (2000).

Finally, to test if the home populations performed better than the foreign populations, we included an additional test for the effect of home vs. away for both datasets.

## Results

The soils from the open environments and from the forests differed significantly in all of the measured characteristics, except for water holding capacity (Table 1). Specifically, the soils from the open environments had higher pH, contained less phosphorus, nitrogen and carbon and had a higher C/N ratio (Table 1). However, the effect of locality was equally as strong as the effect of environment type and was significant for all of the measured soil characteristics (Table 1). All the data on plant size in the experiment are given in S1 Table.

### Comparison between open and forest environments (*A*. *ramosum* only)

**Figure 2:**
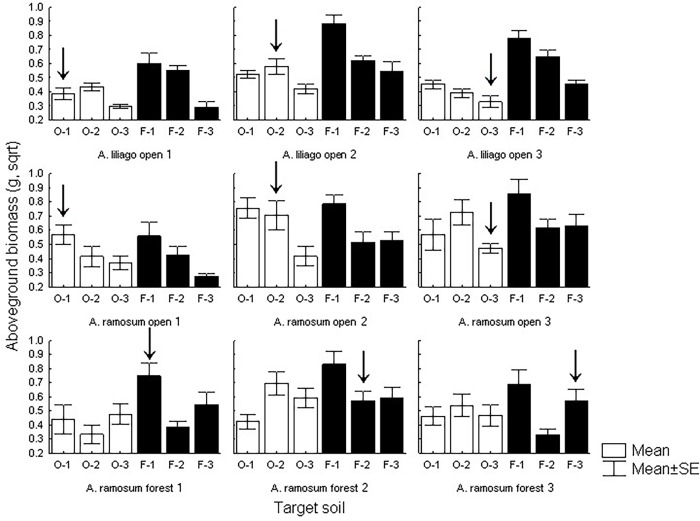
The effect of species, locality of origin and target soil on aboveground biomass. Single panels represent single plant populations (single locality of origin) grown in the common garden. O-1, O-2, O-3, F-1, F-2 and F-3 represent target soils. The target soils either come from open (O, white columns) or forest (F, black columns) environments. The origin and target numbers denote the locality of the population or the soil and correspond to Fig. 1. Arrows indicate plants grown in their home soil.

Aboveground biomass of *A*. *ramosum* from the open and forest environments was strongly affected by target soil and locality of origin. In contrast, neither the target environment nor the environment of origin had a significant effect (Table 2A, Fig. 2). Contrary to our expectations, this result suggests that there is no difference in the aboveground biomass of plants from the open and forest environments and that simulated shading combined with a change in water regime had no significant effect on aboveground plant biomass. The results also indicated that the major factor determining plant aboveground biomass was the soil from which the plant originated and the soil in which the plant was growing. The interaction between target environment and environment of origin was not significant and the interaction between target soil and locality of origin was only marginally significant. Very similar effects were found for flowering probability (Table 2B and 4).

**Table 4:** The effect of target soil and locality of origin on probability of flowering of *A*. *ramosum*.

		Target soil					
		O-1	O-2	O-3	F-1	F-2	F-3
Locality of origin	O-1	0.44	0.20	0.10	0.20	0.00	0.00
	O-2	0.89	0.56	0.20	0.50	0.10	0.00
	O-3	0.50	0.56	0.10	0.50	0.00	0.20
	F-1	0.20	0.00	0.20	0.33	0.00	0.00
	F-2	0.25	0.60	0.50	0.50	0.30	0.20
	F-3	0.56	0.44	0.60	0.50	0.20	0.30

O indicates open locality and F indicates forest locality. 1–3 indicate locality number. For target soils, the codes O-1, O-2, O-3, F-1, F-2 and F-3 represent the locality from which the soil used for plant cultivation was collected. For locality of origin, the codes O-1, O-2, O-3, F-1, F-2 and F-3 represent the locality, from which we collected seeds for the experiment. Bold values show plants grown in their home soil.

**Figure 3:**
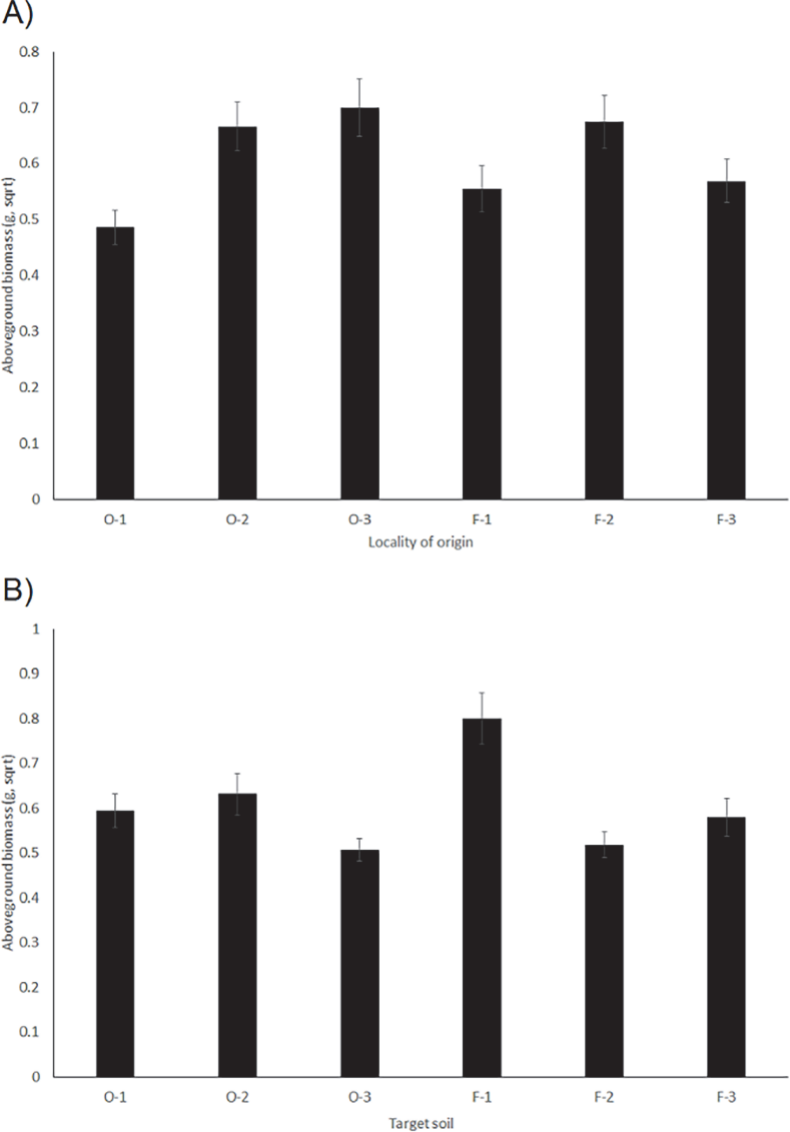
The effect of A) locality of origin and B) target soil on plant aboveground biomass of *A*. *ramosum* in the common garden experiment. O indicates A) open environment of origin and B) soil from open environments, F indicates A) forest environment of origin and B) soil from forest environments. Numbers denote locality numbers and correspond to Fig. 1.

The aboveground biomass of *A*. *ramosum* was significantly lower when the plants originated from the environment with high nitrogen content (localities F-1, F-3 and O-1, Fig. 3A). In addition, the plants grew larger when grown in the soil with high contents of phosphorus, carbon and nitrogen (especially in the F-1 soil, Fig 3B). There was also a significant interaction between the C/N ratio of the environment of origin and the target soil. The plants grew best when they originated from a habitat with a high C/N ratio and were grown in soil with a low C/N ratio (Table 3A, Fig. 2 – most notably population O-3 in F-1 soil).

The probability of flowering in *A*. *ramosum* was more strongly affected by the conditions of the original habitats than those of the target soils. Specifically, the plants flowered more when they originated from conditions of lower pH and carbon content and higher phosphorus content (population F-3, Table 4). The plants also flowered more when grown in soils from open environment with higher pH (soils O-1, O-2 and O-3, Table 4). Similar to aboveground biomass, there was also a significant interaction between the C/N ratio of the locality of origin and the target soil. The plants flowered most when they originated from a locality with a high C/N ratio and were grown in soil with a low C/N ratio (Tables 3B and 4).

The effect of home vs. away was not significant for either aboveground biomass or flowering (p > 0.05 in both cases).

### Comparison between species

**Figure 4:**
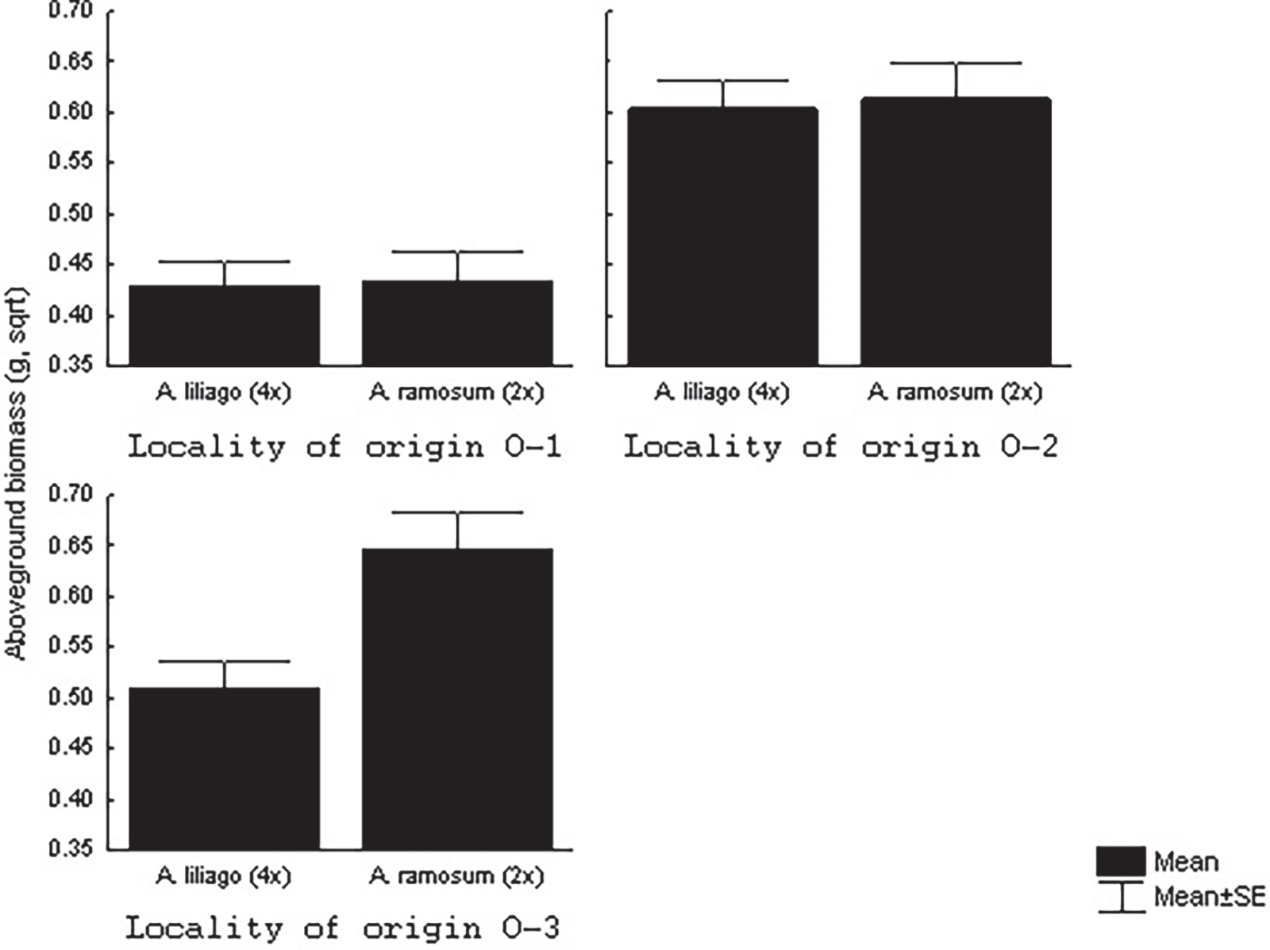
Effects of the locality of origin on the aboveground biomass of *A*. *liliago* and *A*. *ramosum* from the open localities in which the species co-occur. * indicates significant differences between species within the locality.

When comparing *A*. *ramosum* and *A*. *liliago* from the open environment, we also did not find any significant effect of the target environment (simulated open and forest environment) on aboveground plant biomass (Table 2C). As reported above, the results also indicated strong differences in the aboveground biomass of plants grown in different types of target soil (Fig. 2). Plant aboveground biomass also differed between species and localities of origin, and there was a significant interaction between species and locality of origin (Table 2C). Specifically, there was almost no difference in the aboveground biomass of the two species from localities 1 and 2, but the diploid *A*. *ramosum* from locality 3 was significantly larger when compared to the allotetraploid *A*. *liliago* from the same locality (Fig. 4). Locality 3 had significantly lower nitrogen and carbon content and a significantly higher C/N ratio compared to the other localities. The *A*. *ramosum* plants from locality 3 grew the least in their own soil, but the size of the plants was much larger in the more nutrient rich foreign soil (Figs. 2, 4).

The characteristics of the target and origin soils had no significant effects on aboveground biomass in the between-species comparison (p > 0.05 in all cases). Note that there are only three origin soils in this test and the test is thus rather weak. Additionally, the effect of home vs. away and its interaction with species was not significant (p > 0.05).

## Discussion

### The effect of the target environment

We predicted that the target environment will have significant effect on plant performance. Primarily, we expected strong effects of target environment type, i.e., simulated open and forest environments. Thus, it is surprising that the target environment had no significant effects on plant growth and flowering in either of the tests. This result contrasts with numerous published studies suggesting that light availability is a major determinant of plant performance in many systems ([52], [53], [54]). The absence of a shading effect is unlikely due to the low intensity of the shading (a 65% reduction in available light). More likely, it is due to the high variation in soil composition between the localities and thus the strong variation between the different soils within each shading treatment. This suggests that soil composition is more important than shading for the performance of the two species.

The absence of the shading effect on the aboveground biomass of plants is also striking given that the allotetraploid *A*. *liliago* does not usually occur in forested localities; it only rarely forms small populations in open, *Erico-Pinion* forest communities, i.e., open calcareous forests [55]. In addition, we [23] showed strong differences in the population dynamics between diploid *A*. *ramosum* from open and forest environments when studied under natural conditions. The explanation for these differences could include other factors that differentiate the open and forest localities, including differences in microclimatic conditions, differential competition with the surrounding vegetation and the presence of a thick litter layer in the forests. Additionally, it is possible that minority cytotype exclusion plays an important role in restricting tetraploid colonization of the forest localities [56]. Furthermore, the negative effects of shading may be most visible in the early seedling stages. Many studies have, in fact, shown that plants are more sensitive to local habitat conditions in the early stages of development (e.g., [57], [58], [59], [60]). In this experiment, we recorded almost 100% seed germination of the originally sown seeds. Also, almost all experimental plants survived until the end of the experiment. Thus even though we could not study the exact sizes of the plants in early stages due to biology of the plants (many plants retreated early in the first season and reappeared just in the second season), we expect that early performance was not likely different between the plants. Unfortunately, we did not evaluate the belowground biomass of the species at the end of the experiment so we also cannot exclude the possibility that the plants differed in belowground biomass between the two environment types.

In contrast to the absence of significant effects of the target environment, the effect of the target soil was very strong in both comparisons. This aligns with the profound differences in soil chemistry observed between the localities. Similarly large differences in plant performance between the different soil types were previously detected in many different studies (e.g., [33], [61], [62], [63]). In the current study, the importance of local soil conditions is supported by the fact that we were able to detect specific soil elements responsible for plant growth. Specifically, plants grew better in soils with higher carbon, nitrogen and phosphorus contents and flowered more in soils with higher pH. This indicated that they grow better in more nutrient-rich soils. Several recent studies have demonstrated that both abiotic and biotic differences between soils and the interaction between biotic and abiotic factors may drive the response of species to local conditions (e.g., [39], [64], [65]). The assessment of the biotic conditions in the soils was, however, not within the scope of this project.

### The effect of environment of origin

We also predicted that not only the target environment but also the environment of origin will have significant effect on plant performance. Indeed, plant growth was not only affected by the habitat conditions in which they were cultivated but also by the habitat conditions from which they originated. Specifically, plants originating from a system with low nitrogen content and a high C/N ratio grew more. They flowered more when originating from habitats with higher phosphorus content and lower carbon content and pH. A similar effect on offspring performance of nutrient availability to the maternal generation was previously demonstrated in several studies (e.g., [39], [65], [66]).

The strong effect of the environment of origin could have several explanations. One is linked to possible genetic differences between the populations of the species and differential performance between lineages that could be caused by the adaptations of the plants to different environmental conditions (see the discussion below). Alternatively, the differences between the populations could be caused by transgenerational plasticity (maternal effects, e.g., [67], [68], [69]). While the effects of habitat conditions on plant performance in a common garden have between reported in many studies (e.g. [70], [71], [72]), these two alternative explanations are often not considered separately. Thus, both of these possibilities are likely, but distinguishing between them would require that the plants be cultivated over multiple generations (e.g., [73]).

### Differences between the two species and the source environments

Finally, we predicted that response to the environment of origin and the target environment will differ between species. Specifically, we expected that performance of the diploid *A*. *ramosum* from the open environment will not be strongly reduced in simulated forest environment, whereas the allotetraploid *A*. *liliago* will perform worse in the simulated forest environment in which is does not occur in the nature. In addition, we expected that populations of *A*. *ramosum* from different environments will show signs of local adaptation.

Our prediction comparing the two species contrasted to the general expectation for a diploid-polyploid system that the polyploids will have a greater ability to adapt to a wide range of conditions thanks to their increased heterozygosity (e.g., [20]). The results of our study are neither in line with our prediction nor in line with the general expectation as growth of both species across a range of simulated environments suggests that the response of both species to new environmental conditions is very similar. Nevertheless, the diploid *A*. *ramosum* demonstrated a greater ability to adapt to different conditions as plants from nutrient poor habitats clearly had an increased ability to acquire resources. This result supports our hypothesis that *A*. *ramosum* is able to occupy a wider range of habitats thanks to its increased ability to adapt to various conditions. The increased growth in plants from nutrient-poor environments in nutrient-rich environments may either be related to an adaptation of these plants to nutrient shortage that leads to more efficient resource acquisition or to transgenerational plasticity [69].

Even though *A*. *ramosum* shows some signs of adaptation, the plants perform the worst in their home environments, so this is not an indication of local adaptation in a strict sense [29]. A similar pattern, i.e., increased growth in plants from a low nutrient soil transplanted to a high nutrient soil, was also detected by Pankova et al. [74]). In their study, this pattern was explained by higher mycorrhizal root colonization in the plants from the nutrient poor habitat. A similar conclusion, i.e., a significant interaction between habitat conditions in the target environment and the environment of origin but no indication of improved performance in the home localities, was previously reported also for other systems (e.g., [75], and see the review in [76]).

The absence of a home site advantage, and thus the absence of any proof of local adaptation in a strict sense, contrasts with many previous studies that indicate an ability of plants to adapt to their home environments (e.g., [77], [78], [79]). The degree of local adaptation in a system depends on a balance between local selective forces and regional dispersal processes [29]. Gene flow between populations can thus constrain local adaptation to a spatially heterogeneous environment [80]. However, under strong selection, local adaptation may occur despite gene flow from surrounding populations ([81], [82]). Both of the studied species are relatively rare, so their populations are thus quite isolated from each other. In addition, the seeds of both species are quite large and do not have any adaptations for long distance dispersal. All of this suggests that intensive gene flow between the populations is an unlikely explanation for the absence of local adaptation in our system. Alternatively, the absence of local adaptation can be explained by small environmental differences between the studied habitats. While there are large differences in the soil nutrient contents from the different locations, none of these soils are extreme in any way. Previous studies have shown that local adaptation can be found in systems where the local conditions are extreme and can restrict the survival of many species. These conditions could include increased contents of copper, zinc, sodium chloride or magnesium in the soil, herbivory or flooding (e.g., [77], [82], [83], [84], [85]). Other studies have shown that local adaptation is quite common at large spatial scales of several hundreds of kilometers but is less likely at the scale of tens of kilometers (e.g., [75], [86], also see the review in [76]).

Few studies have compared the ability to adapt to local conditions in diploid-polyploid pairs. The studies ([34], [87]) demonstrated a home site advantage in diploid-polyploid systems, but the degree of local adaptation did not differ between the two cytotypes in these studies. Additionally, other reciprocal transplant studies did not find any major differences in the degree of local adaptation between the cytotypes ([35], [36]). Similarly, Hahn et al. [88]) did not find any differences in the response of *Centaurea stoebe* from the native range to environmental conditions.

In line wixth our results, Hulber et al. [89] showed that the performance of diploid *Senecio carniolicus* strongly varied with changing environmental conditions while the performance of hexaploids remained unchanged. Similarly, Schlaepfer et al. [90] showed a significant size response to nutrient treatments in diploid but not tetraploid *Solidago gigantea*. Finally, Buggs & Pannell [31] demonstrated that diploid *Mercurialis annua* was able to grow well in a much wider range of habitat conditions than hexaploid *Mercurialis annua*. These results, i.e., greater morphological changes in diploids compared to polyploids, are similar to the results found in our system and may have two competing explanations. First, the diploids may have higher plasticity and thus a greater ability to adapt to changing conditions (an explanation considered more likely in [31] and [89]). Second, the diploids may, in fact, have a lower ability to adapt to a changing environment, so their growth is reduced in unsuitable conditions. In contrast, polyploids may be more plastic in terms of their metabolism and are thus able to maintain their growth in less suitable conditions (an explanation considered more likely in [90]). As the biomass of diploids increased in some transplant combinations compared to the tetraploids in our experiment, we suggest that the former explanation, i.e., increased ability to adapt to changing conditions, is more likely for diploids in our system.

The above results suggest that *A*. *ramosum* may have a greater ability to acquire limiting resources under some circumstances, which is one possible explanation for its wider distribution. The wider distribution of the diploid may have other explanations as well. Specifically, it may be related to the age of the species as the polyploid taxa is likely younger than the diploid. In support of this, Oberprieler et al. [27] demonstrated that lower ploidy level *Leucanthemum* species occupy a larger proportion of their potential habitats than species with a higher ploidy level. They suggest that species distribution does not reflect the potential ecological advantages associated with polyploidy but rather the age of the taxa because species with lower ploidy levels are expected to be older.

### Conclusions

The study supported the predictions that both the target environment as well as the environment of origin will have strong effects on performance of the two species. Surprisingly, they, however, indicated that the effect of soil conditions of target and origin is more important than the effect of environment type (i.e. simulated forest and open environment). They also supported the prediction that the response to target and origin will differ between the two species. Specifically, the diploid *A*. *ramosum* seems to have the ability to grow in a wider range of conditions thanks to its ability to adapt to changing environments, specifically an increased ability to acquire nutrients when originating from low nutrient environments. In spite of the differences in performance of plants of different origin in the different soils, we did not detect any indication of local adaptation in the system.

## Supporting Information

S1 Table Size of the two *Anthericum* species in the garden experiment. (DOCX)

## References

[1] Soltis DE, Albert VA, Leebens-Mack J, Bell CD, Paterson AH, et al (2009) Polyploidy and Angiosperm diversification. American Journal of Botany 96: 336–348. 10.3732/ajb.0800079 21628192

[2] Levin D (1983) Polyploidy and novelty in flowering plants. American Naturalist 122: 1–25.

[3] Levin D (2002) The Role of Chromosomal Change in Plant Evolution: Oxford Univ Press

[4] Manzaneda AJ, Rey PJ, Bastida JM, Weiss-Lehman C, Raskin E, et al (2012) Environmental aridity is associated with cytotype segregation and polyploidy occurrence in *Brachypodium distachyon* (Poaceae). New Phytologist 193: 797–805. 10.1111/j.1469-8137.2011.03988.x 22150799 PMC3257369

[5] Bretagnolle F, Lumaret R (1995) Bilateral polyploidization in *Dactylis glomerata* L. subsp. *lusitanica*—occurence, morphological and genetic characteristics of first polyploids. Euphytica 84: 197–207.

[6] Nuismer SL, Cunningham BM (2005) Selection for phenotypic divergence between diploid and autotetraploid *Heuchera grossulariifolia* . Evolution 59: 1928–1935. 16261730

[7] Grant V (1994) Modes and origins of mechanical and ethological isolation in Angiosperms. Proceedings of the National Academy of Sciences of the United States of America 91: 3–10. 11607448 10.1073/pnas.91.1.3 PMC42875

[8] Petit C, Lesbros P, Ge XJ, Thompson JD (1997) Variation in flowering phenology and selfing rate across a contact zone between diploid and tetraploid *Arrhenatherum elatius* (Poaceae). Heredity 79: 31–40.

[9] Marques I, Rossello-Graell A, Draper D, Iriondo JM (2007) Pollination patterns limit hybridization between two sympatric species of *Narcissus* (Amaryllidaceae). American Journal of Botany 94: 1352–1359. 10.3732/ajb.94.8.1352 21636503

[10] FelberGirard M, Felber F, Buttler A (1996) Habitat differentiation in a narrow hybrid zone between diploid and tetraploid *Anthoxanthum alpinum* . New Phytologist 133: 531–540.

[11] Parisod C, Holderegger R, Brochmann C (2010) Evolutionary consequences of autopolyploidy. New Phytologist 186: 5–17. 10.1111/j.1469-8137.2009.03142.x 20070540

[12] Bucharova A, Munzbergova Z, Tajek P (2010) Population biology of two rare fern species: long life and long-lasting stability. American Journal of Botany 97: 1260–1271. 10.3732/ajb.0900351 21616878

[13] Bucharova A, Munzbergova Z (2012) Gene Flow among Populations of Two Rare Co-Occurring Fern Species Differing in Ploidy Level. Plos One 7 10.1371/journal.pone.0051204 23029277 PMC3447768

[14] Tajek P, Bucharova A, Muenzbergova Z (2011) Limitation of distribution of two rare ferns in fragmented landscape. Acta Oecologica-International Journal of Ecology 37: 495–502.

[15] Munzbergova Z (2007) No effect of ploidy level in plant response to competition in a common garden experiment. Biological Journal of the Linnean Society 92: 211–219.

[16] Munzbergova Z (2007) Population dynamics of diploid and hexaploid populations of a perennial herb. Annals of Botany 100: 1259–1270. 17881342 10.1093/aob/mcm204 PMC2759246

[17] Castro S, Munzbergova Z, Raabova J, Loureiro J (2011) Breeding barriers at a diploid-hexaploid contact zone in *Aster amellus* . Evolutionary Ecology 25: 795–814.

[18] Jersakova J, Castro S, Sonk N, Milchreit K, Schodelbauerova I, et al (2010) Absence of pollinator-mediated premating barriers in mixed-ploidy populations of *Gymnadenia conopsea* s.l. (Orchidaceae). Evolutionary Ecology 24: 1199–1218.

[19] Godsoe W, Larson MA, Glennon KL, Segraves KA (2013) Polyploidization in *Heuchera cylindrica* (Saxifragaceae) did not result in a shift in climatic requirements. American Journal of Botany 100: 496–508. 10.3732/ajb.1200275 23400493

[20] Eliasova A, Travincek P, Mandak B, Munzbergova Z (2014) Autotetraploids of *Vicia cracca* show a higher allelic richness in natural populations and a higher seed set after artificial selfing than diploids. Annals of Botany 113: 159–170. 10.1093/aob/mct252 24232383 PMC3864723

[21] Soltis DE, Buggs RJA, Doyle JJ, Soltis PS (2010) What we still don't know about polyploidy. Taxon 59: 1387–1403.

[22] Brochmann C, Brysting AK, Alsos IG, Borgen L, Grundt HH, et al (2004) Polyploidy in arctic plants. Biological Journal of the Linnean Society 82: 521–536.

[23] Cerna L, Muenzbergova Z (2013) Comparative Population Dynamics of Two Closely Related Species Differing in Ploidy Level. Plos One 8 10.1371/journal.pone.0082806 24116057 PMC3792132

[24] Meeus S, Honnay O, Jacquemyn H (2012) Strong differences in genetic structure across disjunct, edge and core populations of the distylous forest herb *Pulmonaria officinalis* (Boraginaceae). American Journal of Botany 99: 1809–1818. 10.3732/ajb.1200223 23092991

[25] Eckert CG, Samis KE, Lougheed SC (2008) Genetic variation across species' geographical ranges: the central-marginal hypothesis and beyond. Molecular Ecology 17: 1170–1188. 10.1111/j.1365-294X.2007.03659.x 18302683

[26] Smidova A, Munzbergova Z, Plackova I (2011) Genetic diversity of a relict plant species, *Ligularia sibirica* (L.) Cass. (Asteraceae). Flora 206: 151–157.

[27] Oberprieler C, Konowalik K, Altpeter S, Siegert E, Lo Presti RM, et al (2012) Filling of eco-climatological niches in a polyploid complex—A case study in the plant genus *Leucanthemum* Mill. (Compositae, Anthemideae) from the Iberian Peninsula. Flora 207: 862–867.

[28] Lewis H (1967) The taxonomic significance of autopolyploidy. Taxon 16: 267–271.

[29] Kawecki TJ, Ebert D (2004) Conceptual issues in local adaptation. Ecology Letters 7: 1225–1241.

[30] Hereford J (2009) A Quantitative Survey of Local Adaptation and Fitness Trade-Offs. American Naturalist 173: 579–588. 10.1086/597611 19272016

[31] Buggs RJA, Pannell JR (2007) Ecological differentiation and diploid superiority across a moving ploidy contact zone. Evolution 61: 125–140. 17300432 10.1111/j.1558-5646.2007.00010.x

[32] Laine AL (2007) Detecting local adaptation in a natural plant-pathogen metapopulation: a laboratory vs. field transplant approach. Journal of Evolutionary Biology 20: 1665–1673. 17714283 10.1111/j.1420-9101.2007.01359.x

[33] Raabova J, Munzbergova Z, Fischer M (2011) The role of spatial scale and soil for local adaptation in *Inula hirta* . Basic and Applied Ecology 12: 152–160.

[34] Raabova J, Fischer M, Munzbergova Z (2008) Niche differentiation between diploid and hexaploid *Aster amellus* . Oecologia 158: 463–472. 10.1007/s00442-008-1156-1 18820950

[35] Baack EJ, Stanton ML (2005) Ecological factors influencing tetraploid speciation in snow buttercups (*Ranunculus adoneus*): Niche differentiation and tetraploid establishment. Evolution 59: 1936–1944. 16261731

[36] Flegrova M, Krahulec F (1999) *Anthoxanthum odoratum* and *A*. *alpinum*: Life history parameters at two different altitudes. Folia Geobotanica 34: 19–31.

[37] Rosquist G, Prentice HC (2002) Genetic variation in Scandinavian *Anthericum liliago* (Anthericaceae): allopolyploidy, hybridization and immigration history. Plant Systematics and Evolution 236: 55–72.

[38] Skalický V (1959) Einige taxonomische und phytogeographische Bemerkungen zu den tschechoslovakischen Arten der Gattung *Anthericum* L. Acta Universitatis Carolinae—Biologica 2: 117–157. 20063672

[39] Pankova H, Munzbergova Z, Rydlova J, Vosatka M (2008) Differences in AM fungal root colonization between populations of perennial *Aster* species have genetic reasons. Oecologia 157: 211–220. 10.1007/s00442-008-1064-4 18523810

[40] Olsen R, Cole C, Watanabe F, Dean L (1954) Estimation of available phosphorus in soils by extraction with podium bicarbonate. *US Department of Agriculture Circular* 939: 1–19.

[41] Ehrenberger F, Gorbach S (1973) Methoden der organischen Elementar- und purenanalyse. Weinheim: Verlag Chemie

[42] Munzbergova Z (2004) Effect of spatial scale on factors limiting species distributions in dry grassland fragments. Journal of Ecology 92: 854–867.

[43] Munzbergova Z, Krivanek M, Bucharova A, Juklickova V, Herben T (2005) Ramet performance in two tussock plants—Do the tussock-level parameters matter? Flora 200: 275–284.

[44] Zhang ST, Zhao CA, Lamb EG (2011) Cotyledon damage affects seed number through final plant size in the annual grassland species *Medicago lupulina* . Annals of Botany 107: 437–442. 10.1093/aob/mcq259 21196450 PMC3043934

[45] Abela-Hofbauerova I, Muenzbergova Z (2011) Increased performance of *Cirsium arvense* from the invasive range. Flora 206: 1012–1019.

[46] Burkle LA, Irwin RE (2010) Beyond biomass: measuring the effects of community-level nitrogen enrichment on floral traits, pollinator visitation and plant reproduction. Journal of Ecology 98: 705–717.

[47] Heinken-Smidova A, Munzbergova Z (2012) Population Dynamics of the Endangered, Long-Lived Perennial Species, *Ligularia sibirica* . Folia Geobotanica 47: 193–214.

[48] Weiner J, Campbell LG, Pino J, Echarte L (2009) The allometry of reproduction within plant populations. Journal of Ecology 97: 1220–1233.

[49] Martina JP, von Ende CN (2013) Increased spatial dominance in high nitrogen, saturated soil due to clonal architecture plasticity of the invasive wetland plant, P*halaris arundinacea* . Plant Ecology 214: 1443–1453.

[50] D'Hertefeldt T, Enestrom JM, Pettersson LB (2014) Geographic and Habitat Origin Influence Biomass Production and Storage Translocation in the Clonal Plant *Aegopodium podagraria* . Plos One 9: 8. 10.1371/journal.pone.0085407 PMC3888427 24427305

[51] Francis B, Green M, Pazne C (1993) The Statistical System for Generalized Linear Interactive Modelling. Oxford: Clarendon Press

[52] Skalova H (2005) Morphological plasticity of Festuca rubra clones from three neighbouring communities in response to red: Far-red levels. Folia Geobotanica 40: 77–90.

[53] Xu CY, Schooler SS, Van Klinken RD (2012) Differential Influence of Clonal Integration on Morphological and Growth Responses to Light in Two Invasive Herbs. Plos One 7 10.1371/journal.pone.0051204 22558248 PMC3338812

[54] Sterck FJ, Duursma RA, Pearcy RW, Valladares F, Cieslak M, et al (2013) Plasticity influencing the light compensation point offsets the specialization for light niches across shrub species in a tropical forest understorey. Journal of Ecology 101: 971–980.

[55] Ellenberg H (1988) Vegetation Ecology of Central Europe: Cambridge University Press

[56] Husband BC (2000) Constraints on polyploid evolution: a test of the minority cytotype exclusion principle. Proceedings of the Royal Society B-Biological Sciences 267: 217–223. 10714875 10.1098/rspb.2000.0990 PMC1690524

[57] Freville H, Colas B, Riba M, Caswell H, Mignot A, et al (2004) Spatial and temporal demographic variability in the endemic plant species *Centaurea corymbosa* (Asteraceae). Ecology 85: 694–703.

[58] Munzbergova Z (2005) Determinants of species rarity: Population growth rates of species sharing the same habitat. American Journal of Botany 92: 1987–1994. 10.3732/ajb.92.12.1987 21646117

[59] Dostalek T, Muenzbergova Z (2013) Comparative Population Biology of Critically Endangered *Dracocephalum austriacum* (Lamiaceae) in Two Distant Regions. Folia Geobotanica 48: 75–93.

[60] Muenzbergova Z, Hadincova V, Wild J, Kindlmannova J (2013) Variability in the Contribution of Different Life Stages to Population Growth as a Key Factor in the Invasion Success of Pinus strobus. Plos One 8 10.1371/journal.pone.0082806 23468896 PMC3585251

[61] Raabova J, Muenzbergova Z, Fischer M (2007) Ecological rather than geographic or genetic distance affects local adaptation of the rare perennial herb, *Aster amellus* . Biological Conservation 139: 348–357.

[62] Reckinger C, Colling G, Matthies D (2010) Restoring Populations of the Endangered Plant *Scorzonera humilis*: Influence of Site Conditions, Seed Source, and Plant Stage. Restoration Ecology 18: 904–913.

[63] Lankau RA (2013) Species invasion alters local adaptation to soil communities in a native plant. Ecology 94: 32–40. 23600238 10.1890/12-0675.1

[64] Bever JD, Platt TG, Morton ER (2012) Microbial Population and Community Dynamics on Plant Roots and Their Feedbacks on Plant Communities. Annual Review of Microbiology, Vol 66 66: 265–283. 10.1146/annurev-micro-092611-150107 22726216 PMC3525954

[65] Pankova H, Munzbergova Z, Rydlova J, Vosatka M (2011) The response of *Aster amellus* (Asteraceae) to mycorrhiza depends on the origins of both the soil and the fungi. American Journal of Botany 98: 850–858. 10.3732/ajb.0900350 21613062

[66] Oleksyn J, Reich PB, Zytkowiak R, Karolewski P, Tjoelker MG (2003) Nutrient conservation increases with latitude of origin in European *Pinus sylvestris* populations. Oecologia 136: 220–235. 12756524 10.1007/s00442-003-1265-9

[67] Allen RM, Buckley YM, Marshall DJ (2008) Offspring size plasticity in response to intraspecific competition: An adaptive maternal effect across life-history stages. American Naturalist 171: 225–237. 10.1086/524952 18197775

[68] Latzel V, Hajek T, Klimesova J, Gomez S (2009) Nutrients and disturbance history in two *Plantago* species: maternal effects as a clue for observed dichotomy between resprouting and seeding strategies. Oikos 118: 1669–1678.

[69] Latzel V, Klimesova J, Hajek T, Gomez S, Smilauer P (2010) Maternal effects alter progeny's response to disturbance and nutrients in two *Plantago* species. Oikos 119: 1700–1710.

[70] Munzbergova Z, Plackova I (2010) Seed mass and population characteristics interact to determine performance of *Scorzonera hispanica* under common garden conditions. Flora 205: 552–559.

[71] Lembicz M, Olejniczak P, Zukowski W, Bogdanowicz AM (2011) Effect of mother plant age on germination and size of seeds and seedlings in the perennial sedge *Carex secalina* (Cyperaceae). Flora 206: 158–163.

[72] Chun YJ, Le Corre V, Bretagnolle F (2011) Adaptive divergence for a fitness-related trait among invasive *Ambrosia artemisiifolia* populations in France. Molecular Ecology 20: 1378–1388. 10.1111/j.1365-294X.2011.05013.x 21306459

[73] Jolivet C, Bernasconi G (2007) Molecular and quantitative genetic differentiation in European populations of *Silene latifolia* (Caryophyllaceae). Annals of Botany 100: 119–127. 17565967 10.1093/aob/mcm088 PMC2735300

[74] Pankova H, Raabova J, Muenzbergova Z (2014) Mycorrhizal Symbiosis and Local Adaptation in *Aster amellus*: A Field Transplant Experiment. Plos One 9 10.1371/journal.pone.0115916 24709748 PMC3977983

[75] Macel M, Lawson CS, Mortimer SR, Smilauerova M, Bischoff A, et al (2007) Climate vs. soil factors in local adaptation of two common plant species. Ecology 88: 424–433. 17479760 10.1890/0012-9658(2007)88[424:cvsfil]2.0.co;2

[76] Leimu R, Fischer M (2008) A Meta-Analysis of Local Adaptation in Plants. Plos One 3 10.1371/journal.pone.0002018 19104660 PMC2602971

[77] Wright JW, Stanton ML, Scherson R (2006) Local adaptation to serpentine and non-serpentine soils in *Collinsia sparsiflora* . Evolutionary Ecology Research 8: 1–21.

[78] Weisshuhn K, Prati D, Fischer M, Auge H (2012) Regional adaptation improves the performance of grassland plant communities. Basic and Applied Ecology 13: 551–559.

[79] Bischoff A, Hurault B (2013) Scales and drivers of local adaptation in *Brassica nigra* (Brassicaceae) populations. American Journal of Botany 100: 1162–1170. 10.3732/ajb.1200500 23720429

[80] Slatkin M (1987) Gene flow and the geographic structure of natural populations. Science 236: 787–792. 3576198 10.1126/science.3576198

[81] Jain S, Bradshaw A (1966) Evolutionary divergence aminy adjacent plant populations. 1. The evidence and its theoretical analysis. Heredity 21: 407–441.

[82] McNeilly T, Bradshaw AD (1968) Evolutionary processes in populations of copper tolerant *Agrostis tenuis* Sibth. Evolution 22: 108–&. 28564994 10.1111/j.1558-5646.1968.tb03454.x

[83] Symeonidis L, McNeilly T, Bradshaw AD (1985) Interpopulation variation in tolerance to cadmium, copper, lead, nickel and zinc in 9 populations of *Agrostis capillaris* L. New Phytologist 101: 317–324.

[84] van Kleunen M, Lenssen JPM, Fischer M, de Kroon H (2007) Selection on phenotypic plasticity of morphological traits in response to flooding and competition in the clonal shore plant Ranunculus reptans. Journal of Evolutionary Biology 20: 2126–2137. 17903188 10.1111/j.1420-9101.2007.01431.x

[85] Lenssen JPM, Van Kleunen M, Fischer M, De Kroon H (2004) Local adaptation of the clonal plant *Ranunculus reptans* to flooding along a small-scale gradient. Journal of Ecology 92: 696–706.

[86] Galloway LF, Fenster CB (2000) Population differentiation in an annual legume: Local adaptation. Evolution 54: 1173–1181. 11005286 10.1111/j.0014-3820.2000.tb00552.x

[87] Martin SL, Husband BC (2013) Adaptation of diploid and tetraploid *Chamerion angustifolium* to elevation but not local environment. Evolution 67: 1780–1791. 10.1111/evo.12065 23730769

[88] Hahn MA, van Kleunen M, Muller-Scharer H (2012) Increased Phenotypic Plasticity to Climate May Have Boosted the Invasion Success of Polyploid *Centaurea stoebe* . Plos One 7 10.1371/journal.pone.0051204 23185598 PMC3502303

[89] Hulber K, Berger A, Gilli C, Hofbauer M, Patek M, et al (2011) No evidence for a role of competitive capabilities of adults in causing habitat segregation of diploid and hexaploid *Senecio carniolicus* (Asteracaeae). Alpine Botany 121: 123–127. 10.1007/s00035-011-0091-7 PMC3859894 24348456

[90] Schlaepfer DR, Edwards PJ, Billeter R (2010) Why only tetraploid *Solidago gigantea* (Asteraceae) became invasive: a common garden comparison of ploidy levels. Oecologia 163: 661–673. 10.1007/s00442-010-1595-3 20238128

