# Supplementary material for: Conditions in Home and Transplant Soils Have Differential Effects on the Performance of Diploid and Allotetraploid Anthericum Species
Source: PLoS One. 2015 Jan 21;10(1):e0116992. doi: 10.1371/journal.pone.0116992 (PMC4301807; doi:10.1371/journal.pone.0116992)
Supplement: S1 Table — (DOCX) [file pone.0116992.s001.docx]

Supporting Information Table S1. Size of the two *Anthericum* species in the garden experiment.

| Species | Locality | Population | Soil | Home | Aboveground biomass | Flowering |
| --- | --- | --- | --- | --- | --- | --- |
| 1 | 1 | 1 | 1 | 1 | 0.52 | 0 |
| 1 | 1 | 1 | 1 | 1 | 0.13 | 0 |
| 1 | 1 | 1 | 1 | 1 | 0.12 | 0 |
| 1 | 1 | 1 | 1 | 1 | 0.11 | 0 |
| 1 | 1 | 1 | 1 | 1 | 0.14 | 0 |
| 1 | 1 | 1 | 1 | 1 | 0.09 | 0 |
| 1 | 1 | 1 | 1 | 1 | 0.06 | 0 |
| 1 | 1 | 1 | 1 | 1 | 0.1 | 0 |
| 1 | 1 | 1 | 1 | 1 | 0.27 | 0 |
| 1 | 1 | 1 | 1 | 1 | 0.13 | 0 |
| 2 | 1 | 2 | 1 | 1 |  |  |
| 2 | 1 | 2 | 1 | 1 | 0.37 | 1 |
| 2 | 1 | 2 | 1 | 1 | 0.12 | 0 |
| 2 | 1 | 2 | 1 | 1 | 0.56 | 1 |
| 2 | 1 | 2 | 1 | 1 | 0.17 | 0 |
| 2 | 1 | 2 | 1 | 1 | 0.08 | 0 |
| 2 | 1 | 2 | 1 | 1 | 0.24 | 0 |
| 2 | 1 | 2 | 1 | 1 | 0.54 | 1 |
| 2 | 1 | 2 | 1 | 1 | 0.8 | 0 |
| 2 | 1 | 2 | 1 | 1 | 0.39 | 1 |
| 1 | 2 | 3 | 1 | 0 | 0.28 | 0 |
| 1 | 2 | 3 | 1 | 0 | 0.32 | 0 |
| 1 | 2 | 3 | 1 | 0 | 0.44 | 0 |
| 1 | 2 | 3 | 1 | 0 | 0.375 | 0 |
| 1 | 2 | 3 | 1 | 0 | 0.27 | 0 |
| 1 | 2 | 3 | 1 | 0 | 0.222 | 0 |
| 1 | 2 | 3 | 1 | 0 | 0.189 | 0 |
| 1 | 2 | 3 | 1 | 0 |  |  |
| 1 | 2 | 3 | 1 | 0 | 0.17 | 0 |
| 1 | 2 | 3 | 1 | 0 | 0.233 | 0 |
| 2 | 2 | 4 | 1 | 0 | 0.11 | 0 |
| 2 | 2 | 4 | 1 | 0 | 0.256 | 1 |
| 2 | 2 | 4 | 1 | 0 | 0.5 | 1 |
| 2 | 2 | 4 | 1 | 0 | 0.81 | 1 |
| 2 | 2 | 4 | 1 | 0 | 0.88 | 1 |
| 2 | 2 | 4 | 1 | 0 | 0.89 | 1 |
| 2 | 2 | 4 | 1 | 0 | 0.55 | 1 |
| 2 | 2 | 4 | 1 | 0 | 0.64 | 1 |
| 2 | 2 | 4 | 1 | 0 | 0.85 | 1 |
| 2 | 2 | 4 | 1 | 0 |  |  |
| 1 | 3 | 5 | 1 | 0 | 0.25 | 0 |
| 1 | 3 | 5 | 1 | 0 | 0.42 | 0 |
| 1 | 3 | 5 | 1 | 0 | 0.35 | 0 |
| 1 | 3 | 5 | 1 | 0 | 0.16 | 0 |
| 1 | 3 | 5 | 1 | 0 | 0.17 | 0 |
| 1 | 3 | 5 | 1 | 0 | 0.15 | 0 |
| 1 | 3 | 5 | 1 | 0 | 0.15 | 0 |
| 1 | 3 | 5 | 1 | 0 | 0.2 | 0 |
| 1 | 3 | 5 | 1 | 0 | 0.12 | 0 |
| 1 | 3 | 5 | 1 | 0 | 0.18 | 0 |
| 2 | 3 | 6 | 1 | 0 |  |  |
| 2 | 3 | 6 | 1 | 0 | 0.54 | 1 |
| 2 | 3 | 6 | 1 | 0 | 0.36 | 0 |
| 2 | 3 | 6 | 1 | 0 | 0.986 | 1 |
| 2 | 3 | 6 | 1 | 0 | 0.08 | 0 |
| 2 | 3 | 6 | 1 | 0 | 0.26 | 0 |
| 2 | 3 | 6 | 1 | 0 | 0 | 1 |
| 2 | 3 | 6 | 1 | 0 | 0.541 | 1 |
| 2 | 3 | 6 | 1 | 0 | 0.49 | 0 |
| 2 | 3 | 6 | 1 | 0 |  |  |
| 2 | 4 | 7 | 1 | 0 | 0.09 | 0 |
| 2 | 4 | 7 | 1 | 0 | 0.14 | 0 |
| 2 | 4 | 7 | 1 | 0 | 0.41 | 0 |
| 2 | 4 | 7 | 1 | 0 | 0.01 | 0 |
| 2 | 4 | 7 | 1 | 0 | 0.56 | 1 |
| 2 | 4 | 7 | 1 | 0 | 0.54 | 0 |
| 2 | 4 | 7 | 1 | 0 | 0 | 0 |
| 2 | 4 | 7 | 1 | 0 | 0.42 | 0 |
| 2 | 4 | 7 | 1 | 0 | 0.74 | 1 |
| 2 | 4 | 7 | 1 | 0 | 0 | 0 |
| 2 | 5 | 8 | 1 | 0 |  |  |
| 2 | 5 | 8 | 1 | 0 | 0.08 | 1 |
| 2 | 5 | 8 | 1 | 0 | 0.27 | 0 |
| 2 | 5 | 8 | 1 | 0 | 0.34 | 0 |
| 2 | 5 | 8 | 1 | 0 | 0.12 | 0 |
| 2 | 5 | 8 | 1 | 0 | 0.08 | 0 |
| 2 | 5 | 8 | 1 | 0 | 0.4 | 1 |
| 2 | 5 | 8 | 1 | 0 | 0.09 | 0 |
| 2 | 5 | 8 | 1 | 0 | 0.19 | 0 |
| 2 | 5 | 8 | 1 | 0 |  |  |
| 2 | 6 | 9 | 1 | 0 | 0.16 | 1 |
| 2 | 6 | 9 | 1 | 0 | 0.37 | 1 |
| 2 | 6 | 9 | 1 | 0 | 0.27 | 1 |
| 2 | 6 | 9 | 1 | 0 | 0.08 | 0 |
| 2 | 6 | 9 | 1 | 0 | 0.08 | 0 |
| 2 | 6 | 9 | 1 | 0 |  |  |
| 2 | 6 | 9 | 1 | 0 | 0.08 | 0 |
| 2 | 6 | 9 | 1 | 0 | 0.5 | 1 |
| 2 | 6 | 9 | 1 | 0 | 0.1 | 0 |
| 2 | 6 | 9 | 1 | 0 | 0.59 | 1 |
| 1 | 1 | 1 | 2 | 0 | 0.11 | 0 |
| 1 | 1 | 1 | 2 | 0 | 0.23 | 0 |
| 1 | 1 | 1 | 2 | 0 | 0.26 | 0 |
| 1 | 1 | 1 | 2 | 0 | 0.23 | 0 |
| 1 | 1 | 1 | 2 | 0 |  |  |
| 1 | 1 | 1 | 2 | 0 | 0.19 | 0 |
| 1 | 1 | 1 | 2 | 0 |  |  |
| 1 | 1 | 1 | 2 | 0 |  |  |
| 1 | 1 | 1 | 2 | 0 | 0.15 | 0 |
| 1 | 1 | 1 | 2 | 0 |  |  |
| 2 | 1 | 2 | 2 | 0 | 0.52 | 1 |
| 2 | 1 | 2 | 2 | 0 | 0.15 | 0 |
| 2 | 1 | 2 | 2 | 0 | 0.21 | 0 |
| 2 | 1 | 2 | 2 | 0 | 0.68 | 1 |
| 2 | 1 | 2 | 2 | 0 | 0.07 | 0 |
| 2 | 1 | 2 | 2 | 0 | 0 | 0 |
| 2 | 1 | 2 | 2 | 0 | 0.13 | 0 |
| 2 | 1 | 2 | 2 | 0 | 0.1 | 0 |
| 2 | 1 | 2 | 2 | 0 | 0.1 | 0 |
| 2 | 1 | 2 | 2 | 0 | 0.25 | 0 |
| 1 | 2 | 3 | 2 | 1 | 0.54 | 0 |
| 1 | 2 | 3 | 2 | 1 | 0.26 | 0 |
| 1 | 2 | 3 | 2 | 1 | 0.17 | 0 |
| 1 | 2 | 3 | 2 | 1 | 0.84 | 0 |
| 1 | 2 | 3 | 2 | 1 | 0.29 | 0 |
| 1 | 2 | 3 | 2 | 1 |  |  |
| 1 | 2 | 3 | 2 | 1 | 0.51 | 0 |
| 1 | 2 | 3 | 2 | 1 | 0.23 | 0 |
| 1 | 2 | 3 | 2 | 1 | 0.24 | 0 |
| 1 | 2 | 3 | 2 | 1 | 0.19 | 0 |
| 2 | 2 | 4 | 2 | 1 | 0.69 | 1 |
| 2 | 2 | 4 | 2 | 1 | 0.9 | 1 |
| 2 | 2 | 4 | 2 | 1 | 1.01 | 1 |
| 2 | 2 | 4 | 2 | 1 | 0.85 | 1 |
| 2 | 2 | 4 | 2 | 1 | 1.08 | 1 |
| 2 | 2 | 4 | 2 | 1 | 0.25 | 0 |
| 2 | 2 | 4 | 2 | 1 | 0.23 | 0 |
| 2 | 2 | 4 | 2 | 1 | 0.04 | 0 |
| 2 | 2 | 4 | 2 | 1 | 0.17 | 0 |
| 2 | 2 | 4 | 2 | 1 |  |  |
| 1 | 3 | 5 | 2 | 0 | 0.11 | 0 |
| 1 | 3 | 5 | 2 | 0 | 0.11 | 0 |
| 1 | 3 | 5 | 2 | 0 | 0.19 | 0 |
| 1 | 3 | 5 | 2 | 0 | 0.14 | 0 |
| 1 | 3 | 5 | 2 | 0 | 0.11 | 0 |
| 1 | 3 | 5 | 2 | 0 | 0.04 | 0 |
| 1 | 3 | 5 | 2 | 0 | 0.22 | 0 |
| 1 | 3 | 5 | 2 | 0 | 0.24 | 0 |
| 1 | 3 | 5 | 2 | 0 | 0.24 | 0 |
| 1 | 3 | 5 | 2 | 0 | 0.21 | 0 |
| 2 | 3 | 6 | 2 | 0 |  |  |
| 2 | 3 | 6 | 2 | 0 | 0.71 | 1 |
| 2 | 3 | 6 | 2 | 0 | 0.18 | 0 |
| 2 | 3 | 6 | 2 | 0 | 0.68 | 1 |
| 2 | 3 | 6 | 2 | 0 | 0.189 | 0 |
| 2 | 3 | 6 | 2 | 0 | 0.22 | 0 |
| 2 | 3 | 6 | 2 | 0 | 1.22 | 1 |
| 2 | 3 | 6 | 2 | 0 | 0.65 | 1 |
| 2 | 3 | 6 | 2 | 0 | 1.12 | 1 |
| 2 | 3 | 6 | 2 | 0 | 0.31 | 0 |
| 2 | 4 | 7 | 2 | 0 | 0.24 | 0 |
| 2 | 4 | 7 | 2 | 0 | 0.12 | 0 |
| 2 | 4 | 7 | 2 | 0 | 0.13 | 0 |
| 2 | 4 | 7 | 2 | 0 | 0.05 | 0 |
| 2 | 4 | 7 | 2 | 0 | 0.22 | 0 |
| 2 | 4 | 7 | 2 | 0 | 0.19 | 0 |
| 2 | 4 | 7 | 2 | 0 | 0 | 0 |
| 2 | 4 | 7 | 2 | 0 | 0.3 | 0 |
| 2 | 4 | 7 | 2 | 0 | 0.21 | 0 |
| 2 | 4 | 7 | 2 | 0 | 0 | 0 |
| 2 | 5 | 8 | 2 | 0 | 0.18 | 1 |
| 2 | 5 | 8 | 2 | 0 | 0.76 | 1 |
| 2 | 5 | 8 | 2 | 0 | 1.27 | 1 |
| 2 | 5 | 8 | 2 | 0 | 0.28 | 0 |
| 2 | 5 | 8 | 2 | 0 | 0.18 | 0 |
| 2 | 5 | 8 | 2 | 0 | 0.19 | 0 |
| 2 | 5 | 8 | 2 | 0 | 0.75 | 1 |
| 2 | 5 | 8 | 2 | 0 | 0.63 | 1 |
| 2 | 5 | 8 | 2 | 0 | 1.01 | 1 |
| 2 | 5 | 8 | 2 | 0 | 0.23 | 0 |
| 2 | 6 | 9 | 2 | 0 |  |  |
| 2 | 6 | 9 | 2 | 0 | 0.1 | 0 |
| 2 | 6 | 9 | 2 | 0 | 0.18 | 0 |
| 2 | 6 | 9 | 2 | 0 | 0.15 | 1 |
| 2 | 6 | 9 | 2 | 0 | 0.695 | 1 |
| 2 | 6 | 9 | 2 | 0 | 0.06 | 0 |
| 2 | 6 | 9 | 2 | 0 | 0.54 | 1 |
| 2 | 6 | 9 | 2 | 0 | 0.87 | 1 |
| 2 | 6 | 9 | 2 | 0 | 0.21 | 0 |
| 2 | 6 | 9 | 2 | 0 | 0.27 | 0 |
| 1 | 1 | 1 | 3 | 0 | 0.07 | 0 |
| 1 | 1 | 1 | 3 | 0 | 0.1 | 0 |
| 1 | 1 | 1 | 3 | 0 | 0.13 | 0 |
| 1 | 1 | 1 | 3 | 0 | 0.07 | 0 |
| 1 | 1 | 1 | 3 | 0 | 0.07 | 0 |
| 1 | 1 | 1 | 3 | 0 | 0.13 | 0 |
| 1 | 1 | 1 | 3 | 0 | 0.11 | 0 |
| 1 | 1 | 1 | 3 | 0 |  |  |
| 1 | 1 | 1 | 3 | 0 | 0.05 | 0 |
| 1 | 1 | 1 | 3 | 0 | 0.08 | 0 |
| 2 | 1 | 2 | 3 | 0 | 0.2 | 0 |
| 2 | 1 | 2 | 3 | 0 | 0.1 | 0 |
| 2 | 1 | 2 | 3 | 0 | 0.19 | 0 |
| 2 | 1 | 2 | 3 | 0 | 0.51 | 1 |
| 2 | 1 | 2 | 3 | 0 | 0.03 | 0 |
| 2 | 1 | 2 | 3 | 0 | 0.14 | 0 |
| 2 | 1 | 2 | 3 | 0 | 0.11 | 0 |
| 2 | 1 | 2 | 3 | 0 | 0.07 | 0 |
| 2 | 1 | 2 | 3 | 0 | 0.1 | 0 |
| 2 | 1 | 2 | 3 | 0 | 0.11 | 0 |
| 1 | 2 | 3 | 3 | 0 | 0.11 | 0 |
| 1 | 2 | 3 | 3 | 0 | 0.14 | 0 |
| 1 | 2 | 3 | 3 | 0 | 0.12 | 0 |
| 1 | 2 | 3 | 3 | 0 | 0.16 | 0 |
| 1 | 2 | 3 | 3 | 0 | 0.19 | 0 |
| 1 | 2 | 3 | 3 | 0 |  |  |
| 1 | 2 | 3 | 3 | 0 | 0.32 | 0 |
| 1 | 2 | 3 | 3 | 0 | 0.31 | 0 |
| 1 | 2 | 3 | 3 | 0 | 0.13 | 0 |
| 1 | 2 | 3 | 3 | 0 |  |  |
| 2 | 2 | 4 | 3 | 0 | 0.14 | 0 |
| 2 | 2 | 4 | 3 | 0 | 0.15 | 0 |
| 2 | 2 | 4 | 3 | 0 | 0.31 | 0 |
| 2 | 2 | 4 | 3 | 0 | 0.64 | 1 |
| 2 | 2 | 4 | 3 | 0 | 0.09 | 0 |
| 2 | 2 | 4 | 3 | 0 | 0.26 | 0 |
| 2 | 2 | 4 | 3 | 0 | 0.15 | 0 |
| 2 | 2 | 4 | 3 | 0 | 0.33 | 1 |
| 2 | 2 | 4 | 3 | 0 | 0.08 | 0 |
| 2 | 2 | 4 | 3 | 0 | 0 | 0 |
| 1 | 3 | 5 | 3 | 1 | 0.08 | 0 |
| 1 | 3 | 5 | 3 | 1 | 0 | 0 |
| 1 | 3 | 5 | 3 | 1 | 0.12 | 0 |
| 1 | 3 | 5 | 3 | 1 | 0.11 | 0 |
| 1 | 3 | 5 | 3 | 1 | 0.097 | 0 |
| 1 | 3 | 5 | 3 | 1 | 0.14 | 0 |
| 1 | 3 | 5 | 3 | 1 | 0.14 | 0 |
| 1 | 3 | 5 | 3 | 1 | 0.18 | 0 |
| 1 | 3 | 5 | 3 | 1 | 0.12 | 0 |
| 1 | 3 | 5 | 3 | 1 | 0.25 | 0 |
| 2 | 3 | 6 | 3 | 1 | 0.3 | 0 |
| 2 | 3 | 6 | 3 | 1 | 0.18 | 0 |
| 2 | 3 | 6 | 3 | 1 | 0.11 | 0 |
| 2 | 3 | 6 | 3 | 1 | 0.115 | 0 |
| 2 | 3 | 6 | 3 | 1 | 0.29 | 0 |
| 2 | 3 | 6 | 3 | 1 | 0.31 | 0 |
| 2 | 3 | 6 | 3 | 1 | 0.31 | 0 |
| 2 | 3 | 6 | 3 | 1 | 0.11 | 0 |
| 2 | 3 | 6 | 3 | 1 | 0.33 | 1 |
| 2 | 3 | 6 | 3 | 1 | 0.29 | 0 |
| 2 | 4 | 7 | 3 | 0 | 0.14 | 0 |
| 2 | 4 | 7 | 3 | 0 | 0.3 | 0 |
| 2 | 4 | 7 | 3 | 0 | 0.22 | 0 |
| 2 | 4 | 7 | 3 | 0 | 0 | 0 |
| 2 | 4 | 7 | 3 | 0 | 0.28 | 0 |
| 2 | 4 | 7 | 3 | 0 | 0.55 | 1 |
| 2 | 4 | 7 | 3 | 0 | 0.62 | 1 |
| 2 | 4 | 7 | 3 | 0 | 0.1 | 0 |
| 2 | 4 | 7 | 3 | 0 | 0.36 | 0 |
| 2 | 4 | 7 | 3 | 0 | 0.17 | 0 |
| 2 | 5 | 8 | 3 | 0 | 0.48 | 1 |
| 2 | 5 | 8 | 3 | 0 | 0.23 | 0 |
| 2 | 5 | 8 | 3 | 0 | 0.17 | 1 |
| 2 | 5 | 8 | 3 | 0 | 0.09 | 0 |
| 2 | 5 | 8 | 3 | 0 | 0.22 | 0 |
| 2 | 5 | 8 | 3 | 0 | 0.91 | 1 |
| 2 | 5 | 8 | 3 | 0 | 0.24 | 0 |
| 2 | 5 | 8 | 3 | 0 | 0.5 | 1 |
| 2 | 5 | 8 | 3 | 0 | 0.27 | 0 |
| 2 | 5 | 8 | 3 | 0 | 0.79 | 1 |
| 2 | 6 | 9 | 3 | 0 | 0.61 | 1 |
| 2 | 6 | 9 | 3 | 0 | 0.4 | 1 |
| 2 | 6 | 9 | 3 | 0 | 0.39 | 1 |
| 2 | 6 | 9 | 3 | 0 | 0.03 | 0 |
| 2 | 6 | 9 | 3 | 0 | 0.55 | 1 |
| 2 | 6 | 9 | 3 | 0 | 0.3 | 1 |
| 2 | 6 | 9 | 3 | 0 | 0.07 | 0 |
| 2 | 6 | 9 | 3 | 0 | 0.01 | 0 |
| 2 | 6 | 9 | 3 | 0 | 0.27 | 1 |
| 2 | 6 | 9 | 3 | 0 | 0.08 | 0 |
| 1 | 1 | 1 | 4 | 0 | 0.32 | 0 |
| 1 | 1 | 1 | 4 | 0 | 0.19 | 0 |
| 1 | 1 | 1 | 4 | 0 | 1.04 | 0 |
| 1 | 1 | 1 | 4 | 0 | 0.35 | 0 |
| 1 | 1 | 1 | 4 | 0 | 0.75 | 0 |
| 1 | 1 | 1 | 4 | 0 | 0.45 | 0 |
| 1 | 1 | 1 | 4 | 0 | 0.09 | 0 |
| 1 | 1 | 1 | 4 | 0 | 0.19 | 0 |
| 1 | 1 | 1 | 4 | 0 | 0.7 | 0 |
| 1 | 1 | 1 | 4 | 0 | 0.08 | 0 |
| 2 | 1 | 2 | 4 | 0 | 0.49 | 0 |
| 2 | 1 | 2 | 4 | 0 | 0.4 | 0 |
| 2 | 1 | 2 | 4 | 0 | 0.13 | 0 |
| 2 | 1 | 2 | 4 | 0 | 0.19 | 0 |
| 2 | 1 | 2 | 4 | 0 | 1.25 | 1 |
| 2 | 1 | 2 | 4 | 0 | 0.02 | 0 |
| 2 | 1 | 2 | 4 | 0 | 0.61 | 1 |
| 2 | 1 | 2 | 4 | 0 | 0.16 | 0 |
| 2 | 1 | 2 | 4 | 0 | 0.67 | 0 |
| 2 | 1 | 2 | 4 | 0 | 0.04 | 0 |
| 1 | 2 | 3 | 4 | 0 | 0.48 | 0 |
| 1 | 2 | 3 | 4 | 0 | 0.5 | 0 |
| 1 | 2 | 3 | 4 | 0 | 0.77 | 0 |
| 1 | 2 | 3 | 4 | 0 | 1.11 | 0 |
| 1 | 2 | 3 | 4 | 0 | 1.11 | 0 |
| 1 | 2 | 3 | 4 | 0 | 0.59 | 0 |
| 1 | 2 | 3 | 4 | 0 | 1.36 | 1 |
| 1 | 2 | 3 | 4 | 0 | 1.28 | 0 |
| 1 | 2 | 3 | 4 | 0 | 0.49 | 0 |
| 1 | 2 | 3 | 4 | 0 | 0.5 | 0 |
| 2 | 2 | 4 | 4 | 0 | 0.73 | 0 |
| 2 | 2 | 4 | 4 | 0 | 0.84 | 1 |
| 2 | 2 | 4 | 4 | 0 | 0.87 | 1 |
| 2 | 2 | 4 | 4 | 0 | 1.32 | 1 |
| 2 | 2 | 4 | 4 | 0 | 0.21 | 0 |
| 2 | 2 | 4 | 4 | 0 | 0.6 | 0 |
| 2 | 2 | 4 | 4 | 0 | 0.7 | 1 |
| 2 | 2 | 4 | 4 | 0 | 0.44 | 1 |
| 2 | 2 | 4 | 4 | 0 | 0.33 | 0 |
| 2 | 2 | 4 | 4 | 0 | 0.5 | 0 |
| 1 | 3 | 5 | 4 | 0 | 1.1 | 0 |
| 1 | 3 | 5 | 4 | 0 | 0.94 | 0 |
| 1 | 3 | 5 | 4 | 0 | 0.23 | 0 |
| 1 | 3 | 5 | 4 | 0 | 0.76 | 0 |
| 1 | 3 | 5 | 4 | 0 | 0.65 | 0 |
| 1 | 3 | 5 | 4 | 0 | 0.54 | 0 |
| 1 | 3 | 5 | 4 | 0 | 0.89 | 0 |
| 1 | 3 | 5 | 4 | 0 | 0.24 | 0 |
| 1 | 3 | 5 | 4 | 0 | 0.61 | 0 |
| 1 | 3 | 5 | 4 | 0 | 0.4 | 0 |
| 2 | 3 | 6 | 4 | 0 | 0.82 | 1 |
| 2 | 3 | 6 | 4 | 0 | 0.24 | 0 |
| 2 | 3 | 6 | 4 | 0 | 0.91 | 0 |
| 2 | 3 | 6 | 4 | 0 | 0.92 | 1 |
| 2 | 3 | 6 | 4 | 0 | 0.12 | 0 |
| 2 | 3 | 6 | 4 | 0 | 1.44 | 1 |
| 2 | 3 | 6 | 4 | 0 | 1.01 | 1 |
| 2 | 3 | 6 | 4 | 0 | 1.96 | 1 |
| 2 | 3 | 6 | 4 | 0 | 0.5 | 0 |
| 2 | 3 | 6 | 4 | 0 | 0.36 | 0 |
| 2 | 4 | 7 | 4 | 1 |  |  |
| 2 | 4 | 7 | 4 | 1 | 0.04 | 0 |
| 2 | 4 | 7 | 4 | 1 | 0.96 | 1 |
| 2 | 4 | 7 | 4 | 1 | 0.34 | 0 |
| 2 | 4 | 7 | 4 | 1 | 0.55 | 0 |
| 2 | 4 | 7 | 4 | 1 | 1.39 | 1 |
| 2 | 4 | 7 | 4 | 1 | 0.77 | 1 |
| 2 | 4 | 7 | 4 | 1 | 0.34 | 0 |
| 2 | 4 | 7 | 4 | 1 | 0.67 | 0 |
| 2 | 4 | 7 | 4 | 1 | 0.61 | 0 |
| 2 | 5 | 8 | 4 | 0 | 1.13 | 0 |
| 2 | 5 | 8 | 4 | 0 | 0.75 | 1 |
| 2 | 5 | 8 | 4 | 0 | 0.32 | 0 |
| 2 | 5 | 8 | 4 | 0 | 1.58 | 1 |
| 2 | 5 | 8 | 4 | 0 | 0.12 | 0 |
| 2 | 5 | 8 | 4 | 0 | 0.77 | 1 |
| 2 | 5 | 8 | 4 | 0 | 0.42 | 0 |
| 2 | 5 | 8 | 4 | 0 | 0.81 | 1 |
| 2 | 5 | 8 | 4 | 0 | 0.37 | 0 |
| 2 | 5 | 8 | 4 | 0 | 1.43 | 1 |
| 2 | 6 | 9 | 4 | 0 | 0.35 | 0 |
| 2 | 6 | 9 | 4 | 0 | 0.34 | 0 |
| 2 | 6 | 9 | 4 | 0 | 0.67 | 1 |
| 2 | 6 | 9 | 4 | 0 | 0.86 | 1 |
| 2 | 6 | 9 | 4 | 0 | 0 | 0 |
| 2 | 6 | 9 | 4 | 0 | 1.47 | 1 |
| 2 | 6 | 9 | 4 | 0 | 0.46 | 0 |
| 2 | 6 | 9 | 4 | 0 | 0.4 | 0 |
| 2 | 6 | 9 | 4 | 0 | 0.4 | 1 |
| 2 | 6 | 9 | 4 | 0 | 0.7 | 1 |
| 1 | 1 | 1 | 5 | 0 | 0.25 | 0 |
| 1 | 1 | 1 | 5 | 0 | 0.14 | 0 |
| 1 | 1 | 1 | 5 | 0 | 0.45 | 0 |
| 1 | 1 | 1 | 5 | 0 | 0.39 | 0 |
| 1 | 1 | 1 | 5 | 0 | 0.45 | 0 |
| 1 | 1 | 1 | 5 | 0 | 0.27 | 0 |
| 1 | 1 | 1 | 5 | 0 | 0.33 | 0 |
| 1 | 1 | 1 | 5 | 0 | 0.34 | 0 |
| 1 | 1 | 1 | 5 | 0 | 0.41 | 0 |
| 1 | 1 | 1 | 5 | 0 | 0.11 | 0 |
| 2 | 1 | 2 | 5 | 0 |  |  |
| 2 | 1 | 2 | 5 | 0 | 0.16 | 0 |
| 2 | 1 | 2 | 5 | 0 | 0.31 | 0 |
| 2 | 1 | 2 | 5 | 0 | 0.35 | 0 |
| 2 | 1 | 2 | 5 | 0 | 0.17 | 0 |
| 2 | 1 | 2 | 5 | 0 | 0.27 | 0 |
| 2 | 1 | 2 | 5 | 0 | 0.26 | 0 |
| 2 | 1 | 2 | 5 | 0 | 0 | 0 |
| 2 | 1 | 2 | 5 | 0 | 0.1 | 0 |
| 2 | 1 | 2 | 5 | 0 | 0.28 | 0 |
| 1 | 2 | 3 | 5 | 0 | 0.47 | 0 |
| 1 | 2 | 3 | 5 | 0 | 0.29 | 0 |
| 1 | 2 | 3 | 5 | 0 | 0.5 | 0 |
| 1 | 2 | 3 | 5 | 0 | 0.51 | 0 |
| 1 | 2 | 3 | 5 | 0 | 0.41 | 0 |
| 1 | 2 | 3 | 5 | 0 | 0.31 | 0 |
| 1 | 2 | 3 | 5 | 0 | 0.39 | 0 |
| 1 | 2 | 3 | 5 | 0 | 0.39 | 0 |
| 1 | 2 | 3 | 5 | 0 | 0.15 | 0 |
| 1 | 2 | 3 | 5 | 0 | 0.51 | 0 |
| 2 | 2 | 4 | 5 | 0 | 0.12 | 0 |
| 2 | 2 | 4 | 5 | 0 | 1.07 | 1 |
| 2 | 2 | 4 | 5 | 0 | 0.57 | 0 |
| 2 | 2 | 4 | 5 | 0 | 0.12 | 0 |
| 2 | 2 | 4 | 5 | 0 | 0.081 | 0 |
| 2 | 2 | 4 | 5 | 0 | 0.17 | 0 |
| 2 | 2 | 4 | 5 | 0 | 0.18 | 0 |
| 2 | 2 | 4 | 5 | 0 | 0.07 | 0 |
| 2 | 2 | 4 | 5 | 0 | 0.43 | 0 |
| 2 | 2 | 4 | 5 | 0 | 0.38 | 0 |
| 1 | 3 | 5 | 5 | 0 | 0.46 | 0 |
| 1 | 3 | 5 | 5 | 0 | 0.51 | 0 |
| 1 | 3 | 5 | 5 | 0 | 0.12 | 0 |
| 1 | 3 | 5 | 5 | 0 | 0.4 | 0 |
| 1 | 3 | 5 | 5 | 0 | 0.58 | 0 |
| 1 | 3 | 5 | 5 | 0 | 0.54 | 0 |
| 1 | 3 | 5 | 5 | 0 | 0.23 | 0 |
| 1 | 3 | 5 | 5 | 0 | 0.54 | 0 |
| 1 | 3 | 5 | 5 | 0 | 0.7 | 0 |
| 1 | 3 | 5 | 5 | 0 | 0.35 | 0 |
| 2 | 3 | 6 | 5 | 0 | 0.65 | 0 |
| 2 | 3 | 6 | 5 | 0 | 0.25 | 0 |
| 2 | 3 | 6 | 5 | 0 | 0.82 | 0 |
| 2 | 3 | 6 | 5 | 0 | 0.56 | 0 |
| 2 | 3 | 6 | 5 | 0 | 0.26 | 0 |
| 2 | 3 | 6 | 5 | 0 | 0.53 | 0 |
| 2 | 3 | 6 | 5 | 0 | 0.51 | 0 |
| 2 | 3 | 6 | 5 | 0 | 0.21 | 0 |
| 2 | 3 | 6 | 5 | 0 | 0.24 | 0 |
| 2 | 3 | 6 | 5 | 0 | 0.1 | 0 |
| 2 | 4 | 7 | 5 | 0 | 0.171 | 0 |
| 2 | 4 | 7 | 5 | 0 | 0.13 | 0 |
| 2 | 4 | 7 | 5 | 0 | 0.12 | 0 |
| 2 | 4 | 7 | 5 | 0 | 0.07 | 0 |
| 2 | 4 | 7 | 5 | 0 | 0.234 | 0 |
| 2 | 4 | 7 | 5 | 0 | 0.09 | 0 |
| 2 | 4 | 7 | 5 | 0 | 0.04 | 0 |
| 2 | 4 | 7 | 5 | 0 | 0.32 | 0 |
| 2 | 4 | 7 | 5 | 0 | 0.38 | 0 |
| 2 | 4 | 7 | 5 | 0 | 0.09 | 0 |
| 2 | 5 | 8 | 5 | 1 | 0.53 | 1 |
| 2 | 5 | 8 | 5 | 1 | 0.14 | 0 |
| 2 | 5 | 8 | 5 | 1 | 0.13 | 0 |
| 2 | 5 | 8 | 5 | 1 | 0.14 | 0 |
| 2 | 5 | 8 | 5 | 1 | 0.181 | 0 |
| 2 | 5 | 8 | 5 | 1 | 0.66 | 0 |
| 2 | 5 | 8 | 5 | 1 | 0.67 | 1 |
| 2 | 5 | 8 | 5 | 1 | 0.55 | 0 |
| 2 | 5 | 8 | 5 | 1 | 0.63 | 1 |
| 2 | 5 | 8 | 5 | 1 | 0.09 | 0 |
| 2 | 6 | 9 | 5 | 0 | 0.15 | 1 |
| 2 | 6 | 9 | 5 | 0 | 0.01 | 0 |
| 2 | 6 | 9 | 5 | 0 | 0.05 | 0 |
| 2 | 6 | 9 | 5 | 0 | 0.12 | 1 |
| 2 | 6 | 9 | 5 | 0 | 0.05 | 0 |
| 2 | 6 | 9 | 5 | 0 | 0.09 | 0 |
| 2 | 6 | 9 | 5 | 0 | 0.17 | 0 |
| 2 | 6 | 9 | 5 | 0 | 0.18 | 0 |
| 2 | 6 | 9 | 5 | 0 | 0.13 | 0 |
| 2 | 6 | 9 | 5 | 0 | 0.27 | 0 |
| 1 | 1 | 1 | 6 | 0 | 0.07 | 0 |
| 1 | 1 | 1 | 6 | 0 | 0.14 | 0 |
| 1 | 1 | 1 | 6 | 0 | 0.16 | 0 |
| 1 | 1 | 1 | 6 | 0 | 0.11 | 0 |
| 1 | 1 | 1 | 6 | 0 | 0 | 0 |
| 1 | 1 | 1 | 6 | 0 | 0.11 | 0 |
| 1 | 1 | 1 | 6 | 0 | 0.03 | 0 |
| 1 | 1 | 1 | 6 | 0 | 0.14 | 0 |
| 1 | 1 | 1 | 6 | 0 | 0.17 | 0 |
| 1 | 1 | 1 | 6 | 0 | 0.06 | 0 |
| 2 | 1 | 2 | 6 | 0 | 0.06 | 0 |
| 2 | 1 | 2 | 6 | 0 | 0.06 | 0 |
| 2 | 1 | 2 | 6 | 0 | 0.06 | 0 |
| 2 | 1 | 2 | 6 | 0 | 0.09 | 0 |
| 2 | 1 | 2 | 6 | 0 | 0.11 | 0 |
| 2 | 1 | 2 | 6 | 0 | 0.09 | 0 |
| 2 | 1 | 2 | 6 | 0 | 0.14 | 0 |
| 2 | 1 | 2 | 6 | 0 | 0.04 | 0 |
| 2 | 1 | 2 | 6 | 0 | 0.09 | 0 |
| 2 | 1 | 2 | 6 | 0 | 0.05 | 0 |
| 1 | 2 | 3 | 6 | 0 | 0.37 | 0 |
| 1 | 2 | 3 | 6 | 0 | 0.32 | 0 |
| 1 | 2 | 3 | 6 | 0 | 0.02 | 0 |
| 1 | 2 | 3 | 6 | 0 | 0.33 | 0 |
| 1 | 2 | 3 | 6 | 0 | 0.49 | 0 |
| 1 | 2 | 3 | 6 | 0 | 0.15 | 0 |
| 1 | 2 | 3 | 6 | 0 | 0.37 | 0 |
| 1 | 2 | 3 | 6 | 0 | 0.95 | 0 |
| 1 | 2 | 3 | 6 | 0 | 0.16 | 0 |
| 1 | 2 | 3 | 6 | 0 | 0.24 | 0 |
| 2 | 2 | 4 | 6 | 0 | 0.3 | 0 |
| 2 | 2 | 4 | 6 | 0 | 0.33 | 0 |
| 2 | 2 | 4 | 6 | 0 | 0.66 | 0 |
| 2 | 2 | 4 | 6 | 0 | 0.28 | 0 |
| 2 | 2 | 4 | 6 | 0 | 0.74 | 0 |
| 2 | 2 | 4 | 6 | 0 | 0.19 | 0 |
| 2 | 2 | 4 | 6 | 0 | 0.22 | 0 |
| 2 | 2 | 4 | 6 | 0 | 0.04 | 0 |
| 2 | 2 | 4 | 6 | 0 | 0.32 | 0 |
| 2 | 2 | 4 | 6 | 0 | 0.08 | 0 |
| 1 | 3 | 5 | 6 | 0 | 0.2 | 0 |
| 1 | 3 | 5 | 6 | 0 | 0.18 | 0 |
| 1 | 3 | 5 | 6 | 0 | 0.37 | 0 |
| 1 | 3 | 5 | 6 | 0 | 0.2 | 0 |
| 1 | 3 | 5 | 6 | 0 | 0.22 | 0 |
| 1 | 3 | 5 | 6 | 0 | 0.28 | 0 |
| 1 | 3 | 5 | 6 | 0 | 0.09 | 0 |
| 1 | 3 | 5 | 6 | 0 | 0.23 | 0 |
| 1 | 3 | 5 | 6 | 0 | 0.15 | 0 |
| 1 | 3 | 5 | 6 | 0 | 0.224 | 0 |
| 2 | 3 | 6 | 6 | 0 | 1.22 | 1 |
| 2 | 3 | 6 | 6 | 0 | 0.92 | 0 |
| 2 | 3 | 6 | 6 | 0 | 0.45 | 0 |
| 2 | 3 | 6 | 6 | 0 | 0.35 | 1 |
| 2 | 3 | 6 | 6 | 0 | 0.12 | 0 |
| 2 | 3 | 6 | 6 | 0 | 0.12 | 0 |
| 2 | 3 | 6 | 6 | 0 | 0.48 | 0 |
| 2 | 3 | 6 | 6 | 0 | 0.5 | 0 |
| 2 | 3 | 6 | 6 | 0 | 0.36 | 0 |
| 2 | 3 | 6 | 6 | 0 | 0.09 | 0 |
| 2 | 4 | 7 | 6 | 0 | 0.31 | 0 |
| 2 | 4 | 7 | 6 | 0 | 0.65 | 0 |
| 2 | 4 | 7 | 6 | 0 | 0.15 | 0 |
| 2 | 4 | 7 | 6 | 0 | 1.62 | 0 |
| 2 | 4 | 7 | 6 | 0 | 0.12 | 0 |
| 2 | 4 | 7 | 6 | 0 | 0.23 | 0 |
| 2 | 4 | 7 | 6 | 0 | 0.19 | 0 |
| 2 | 4 | 7 | 6 | 0 | 0.19 | 0 |
| 2 | 4 | 7 | 6 | 0 | 0.1 | 0 |
| 2 | 4 | 7 | 6 | 0 | 0.16 | 0 |
| 2 | 5 | 8 | 6 | 0 | 0.01 | 0 |
| 2 | 5 | 8 | 6 | 0 | 0.31 | 0 |
| 2 | 5 | 8 | 6 | 0 | 0.22 | 0 |
| 2 | 5 | 8 | 6 | 0 | 0.23 | 0 |
| 2 | 5 | 8 | 6 | 0 | 0.51 | 0 |
| 2 | 5 | 8 | 6 | 0 | 0.24 | 0 |
| 2 | 5 | 8 | 6 | 0 | 1.1 | 1 |
| 2 | 5 | 8 | 6 | 0 | 0.409 | 0 |
| 2 | 5 | 8 | 6 | 0 | 0.62 | 1 |
| 2 | 5 | 8 | 6 | 0 | 0.361 | 0 |
| 2 | 6 | 9 | 6 | 1 | 0.04 | 0 |
| 2 | 6 | 9 | 6 | 1 | 0.52 | 1 |
| 2 | 6 | 9 | 6 | 1 | 0.05 | 0 |
| 2 | 6 | 9 | 6 | 1 | 0.71 | 1 |
| 2 | 6 | 9 | 6 | 1 | 0.27 | 0 |
| 2 | 6 | 9 | 6 | 1 | 0.95 | 1 |
| 2 | 6 | 9 | 6 | 1 | 0.14 | 0 |
| 2 | 6 | 9 | 6 | 1 | 0.4 | 0 |
| 2 | 6 | 9 | 6 | 1 | 0.22 | 0 |
| 2 | 6 | 9 | 6 | 1 | 0.6 | 0 |
